# Correction: Early warning for healthcare acquired infections in neonatal care units in a low-resource setting using routinely collected hospital data: The experience from Haiti, 2014–2018

**DOI:** 10.1371/journal.pone.0298987

**Published:** 2024-02-12

**Authors:** Annick Lenglet, Omar Contigiani, Cono Ariti, Estivern Evens, Kessianne Charles, Carl-Frédéric Casimir, Rodnie Senat Delva, Colette Badjo, Harriet Roggeveen, Barbara Pawulska, Kate Clezy, Melissa McRae, Heiman Wertheim, Joost Hopman

The Funding statement is incorrect. The correct statement is:

**Funding:** MSF research is undertaken as part of operations and financial support for this research was absorbed by MSF budgets. MSF provided support for this study in the form of salaries for AL, CA, EE, KG, CFC, RDS, CB, HR, BP, KC, and MM. The specific roles of these authors are articulated in the ‘author contributions’ section. MSF was involved in the study design, data collection and analysis, decision to publish, and preparation of the manuscript. Every stage of research and dissemination also received approval from the MSF (OCA) Research Committee.
