# Supplementary material for: Early warning for healthcare acquired infections in neonatal care units in a low-resource setting using routinely collected hospital data: The experience from Haiti, 2014–2018
Source: PLoS One. 2022 Jun 23;17(6):e0269385. doi: 10.1371/journal.pone.0269385 (PMC9223318; doi:10.1371/journal.pone.0269385)
Supplement: S1 File — (DOCX) [file pone.0269385.s001.docx]

***Supplemental information***

| **S1 Figure**: LO sepsis cases differential outbreak alarm (red triangles) and the smoothed future LO sepsis indicator |
| --- |
| 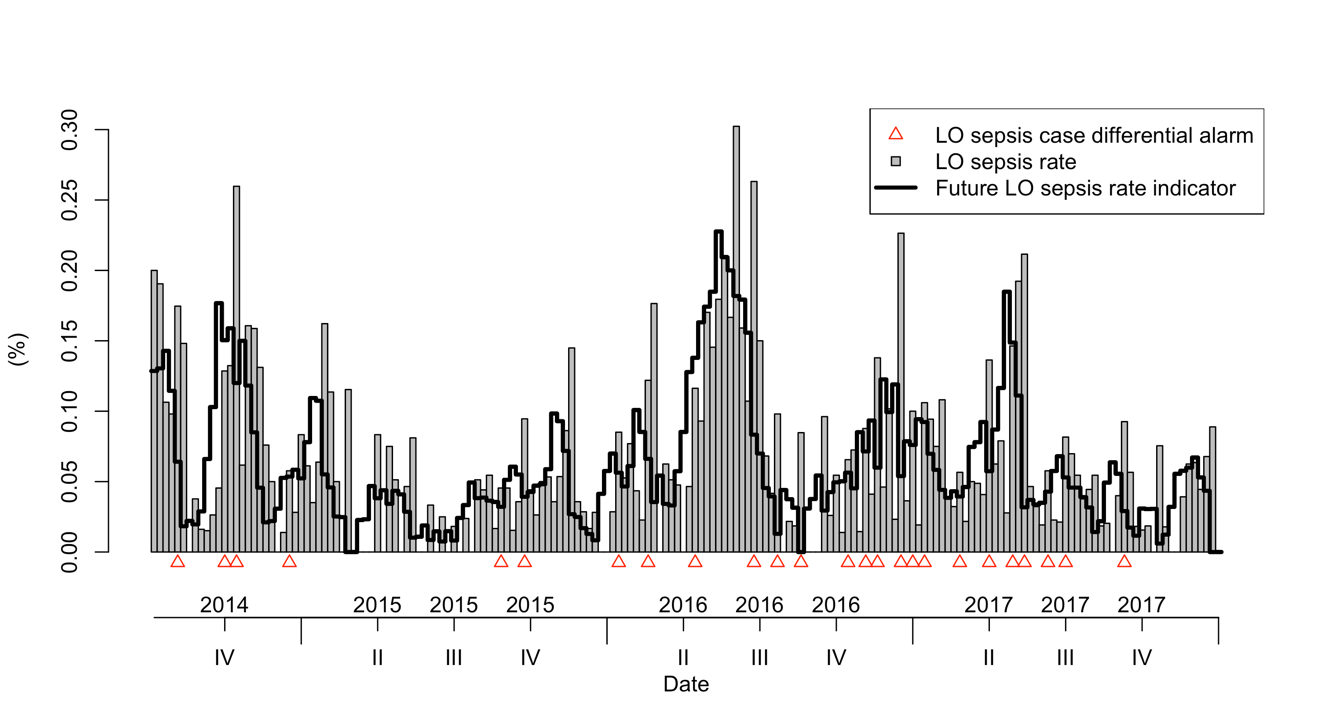 |
| **S2 Figure**: LO sepsis cases differential outbreak alarm (red triangles) and the smoothed future mortality indicator |
| 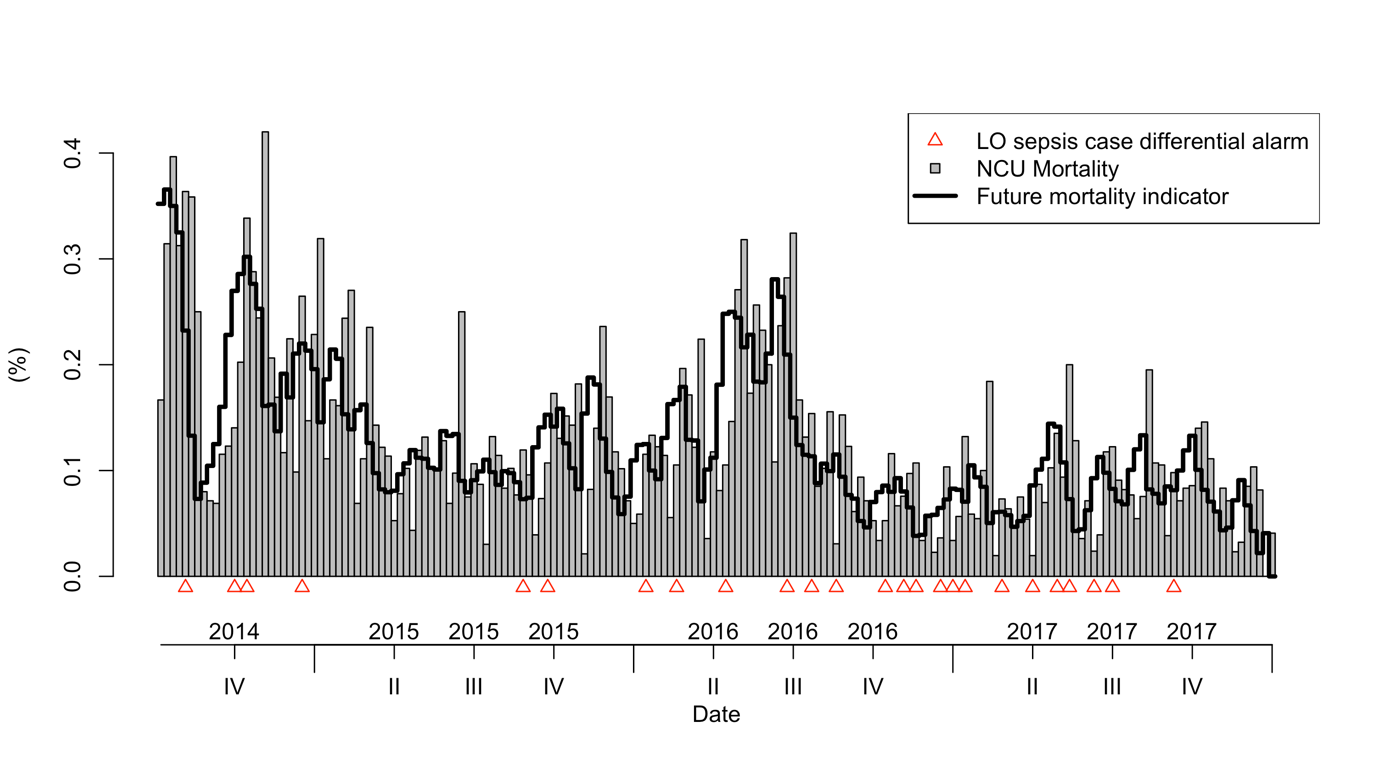 |

| **S3 Figure**: LO sepsis cases differential outbreak alarm (red triangles) and the smoothed GNB positive blood culture indicator |
| --- |
| 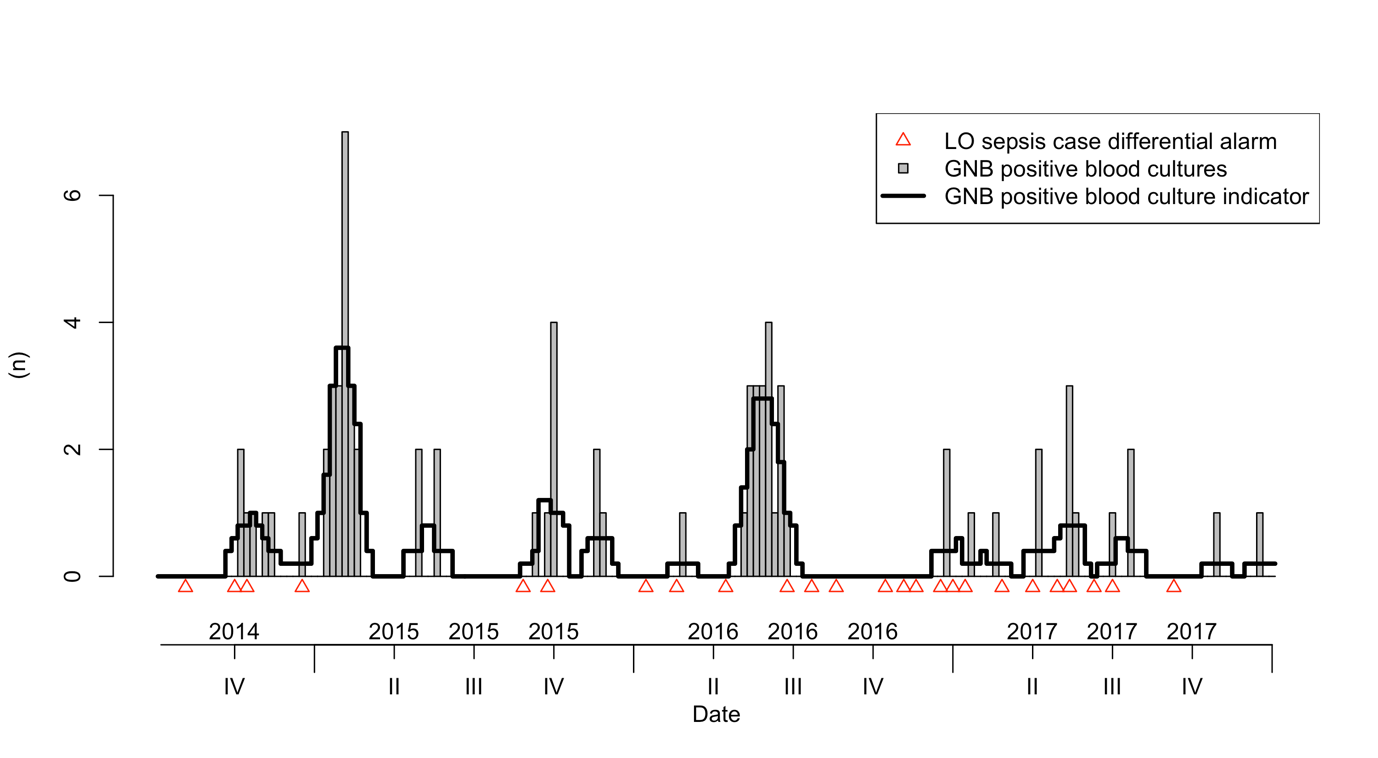 |
| **S4 Figure**: LO sepsis cases differential outbreak alarm (red triangles) and the smoothed LO sepsis rate indicator |
| 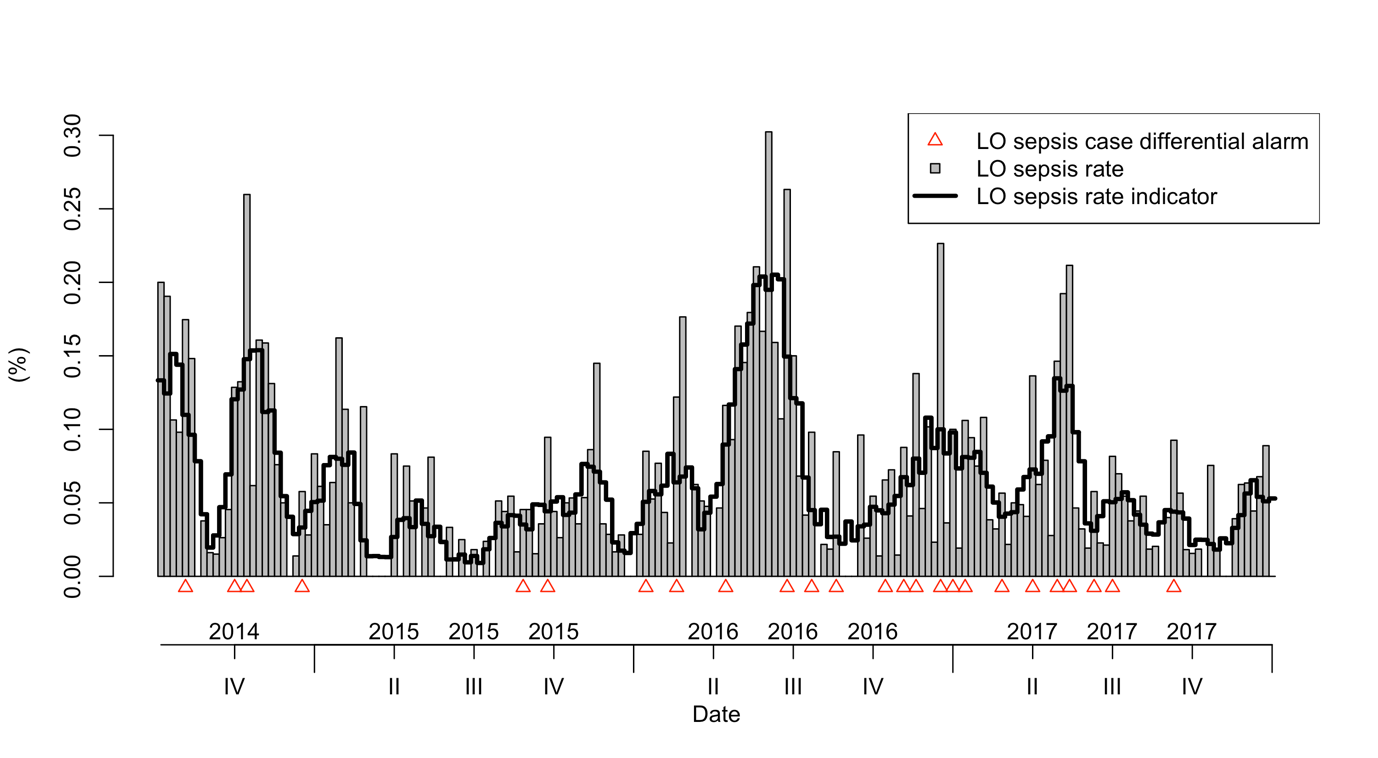 |

| **S5 Figure**: LO sepsis cases differential outbreak alarm (red triangles) and the smoothed NCU mortality indicator |
| --- |
| 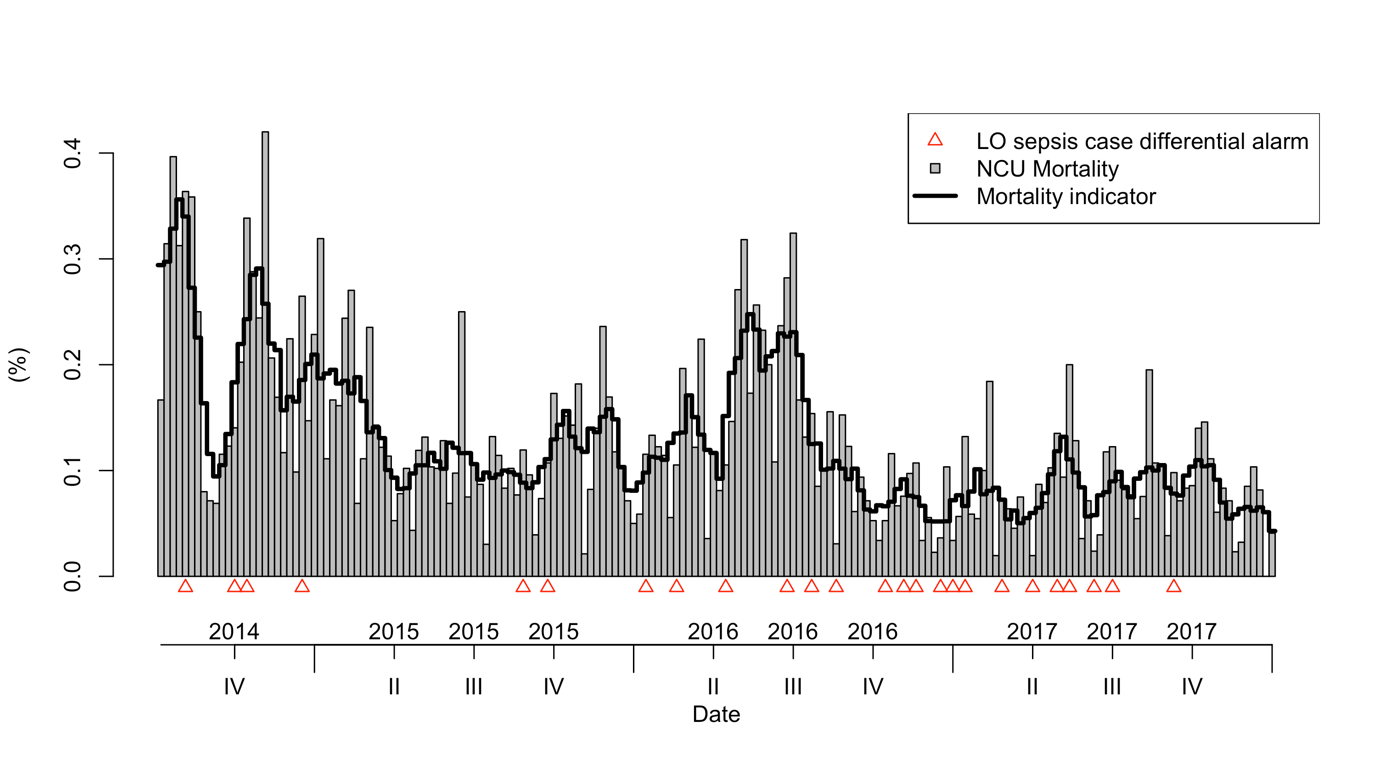 |
| **S6 Figure**: LO sepsis rate aberration alarm (red triangles) and the smoothed future LO sepsis rate indicator |
| 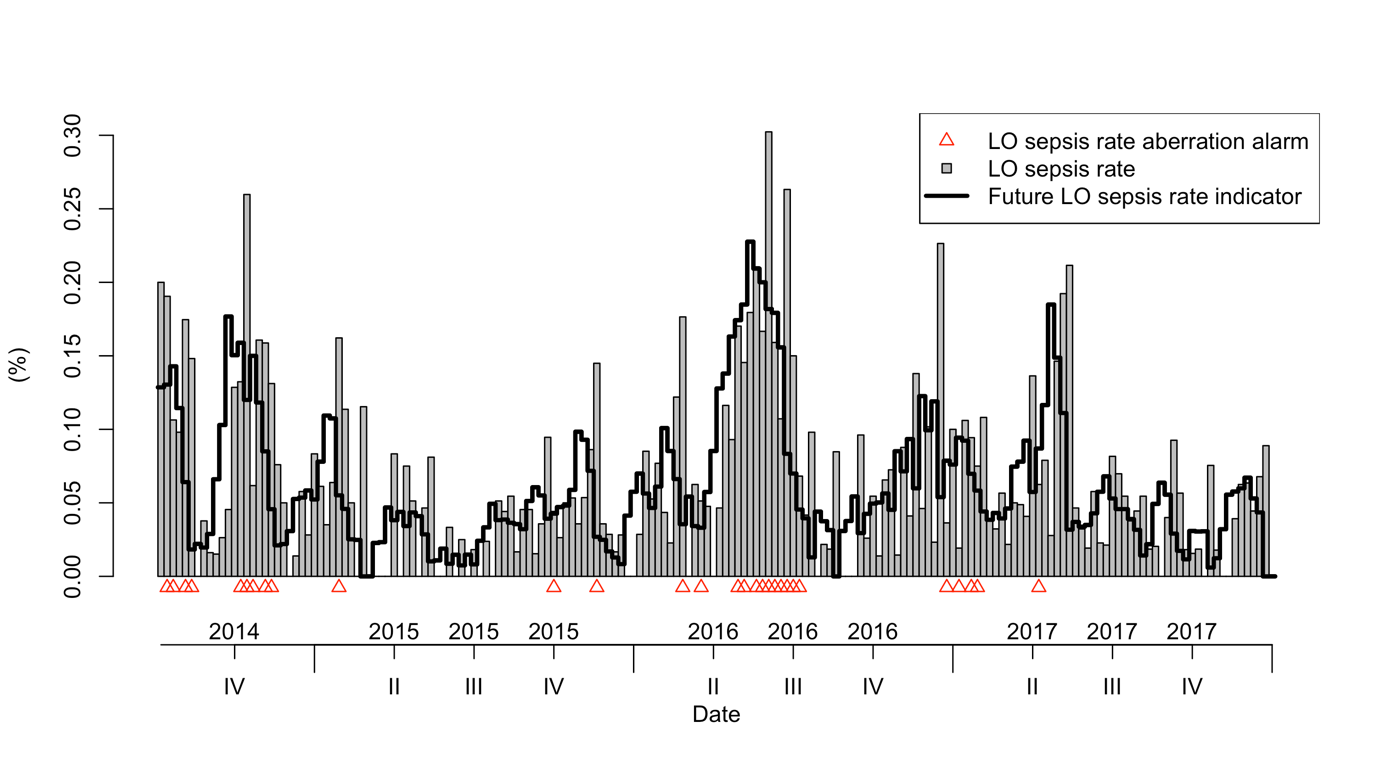 |

| **S7 Figure**: LO sepsis rate aberration alarm (red triangles) and the smoothed future mortality indicator |
| --- |
| 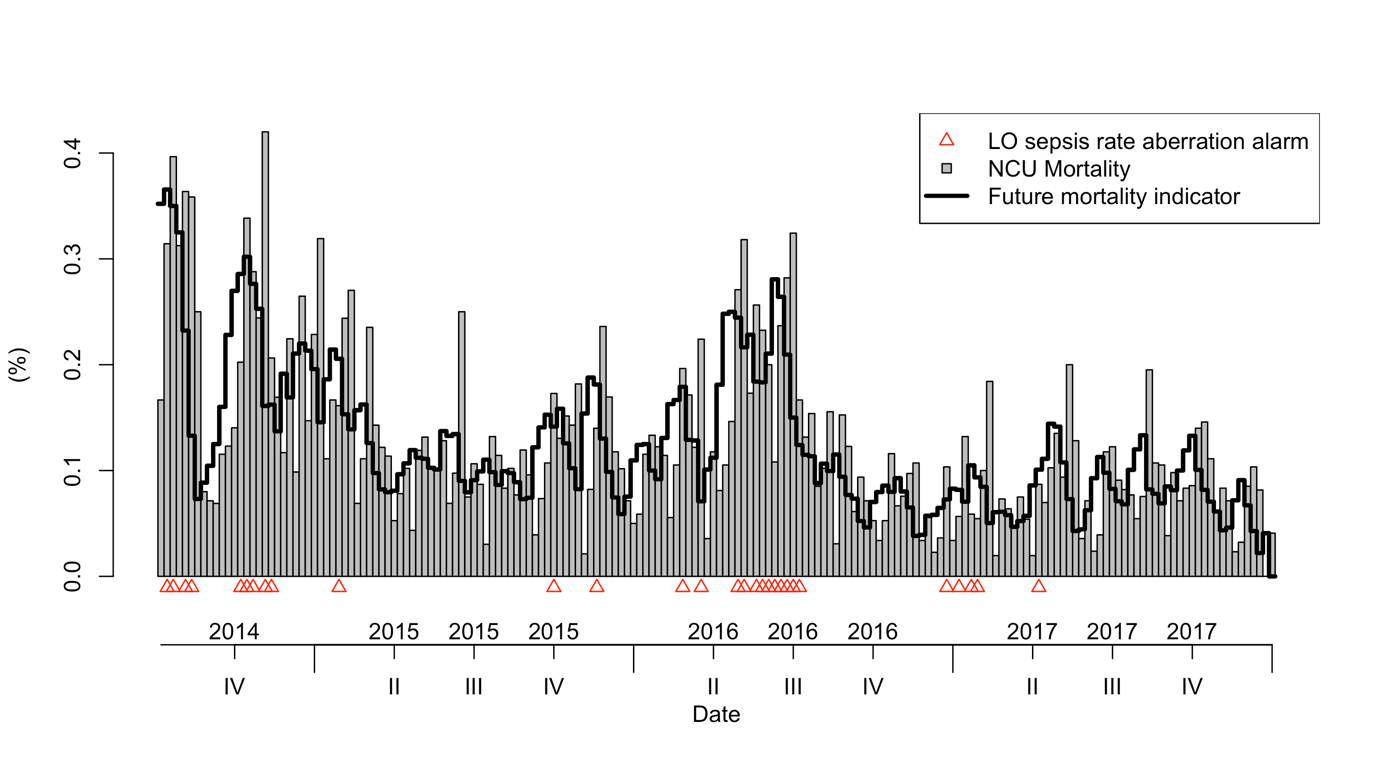 |
| **S8 Figure:** LO sepsis rate aberration alarm (red triangles) and the smoothed GNB positive blood culture indicator |
| 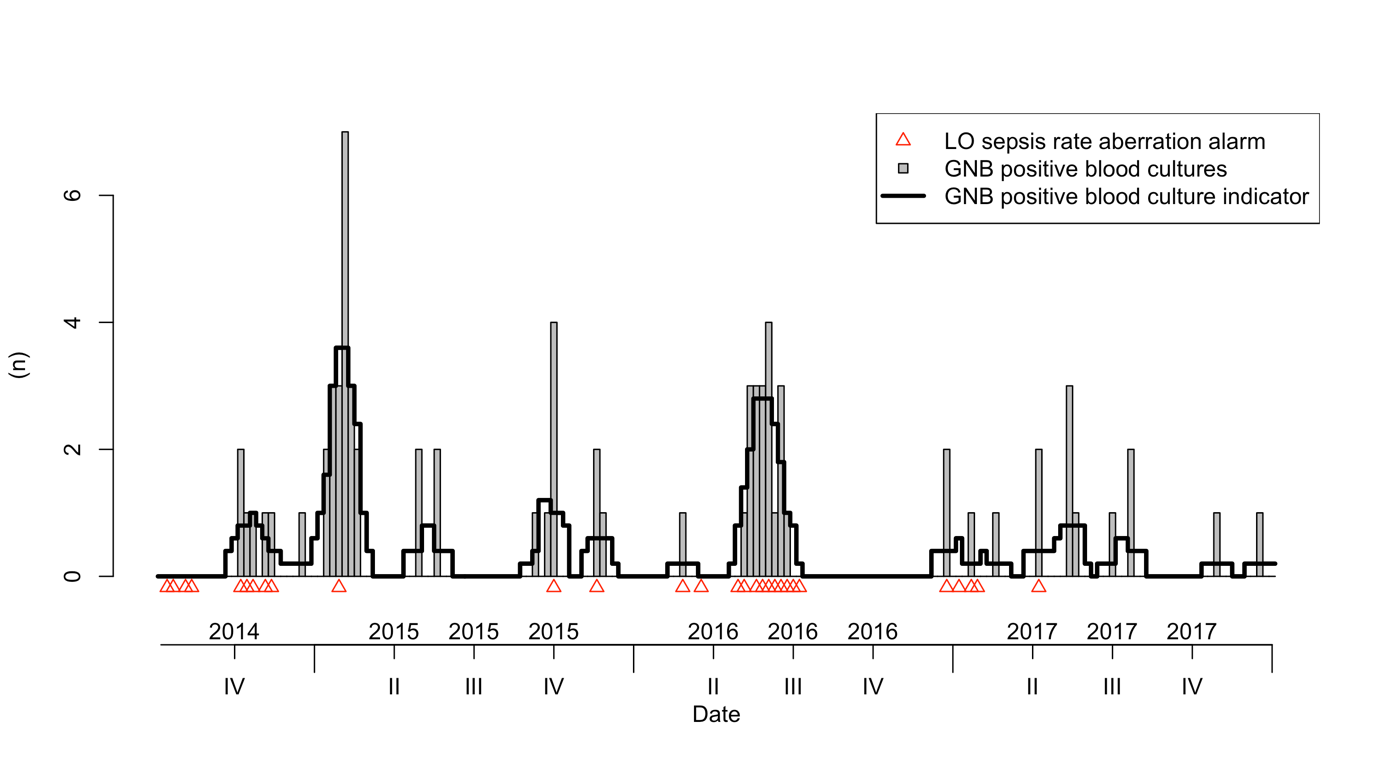 |

| **S9 Figure:** LO sepsis rate threshold alarm (red triangles) and the smoothed future LO sepsis rate indicator |
| --- |
| 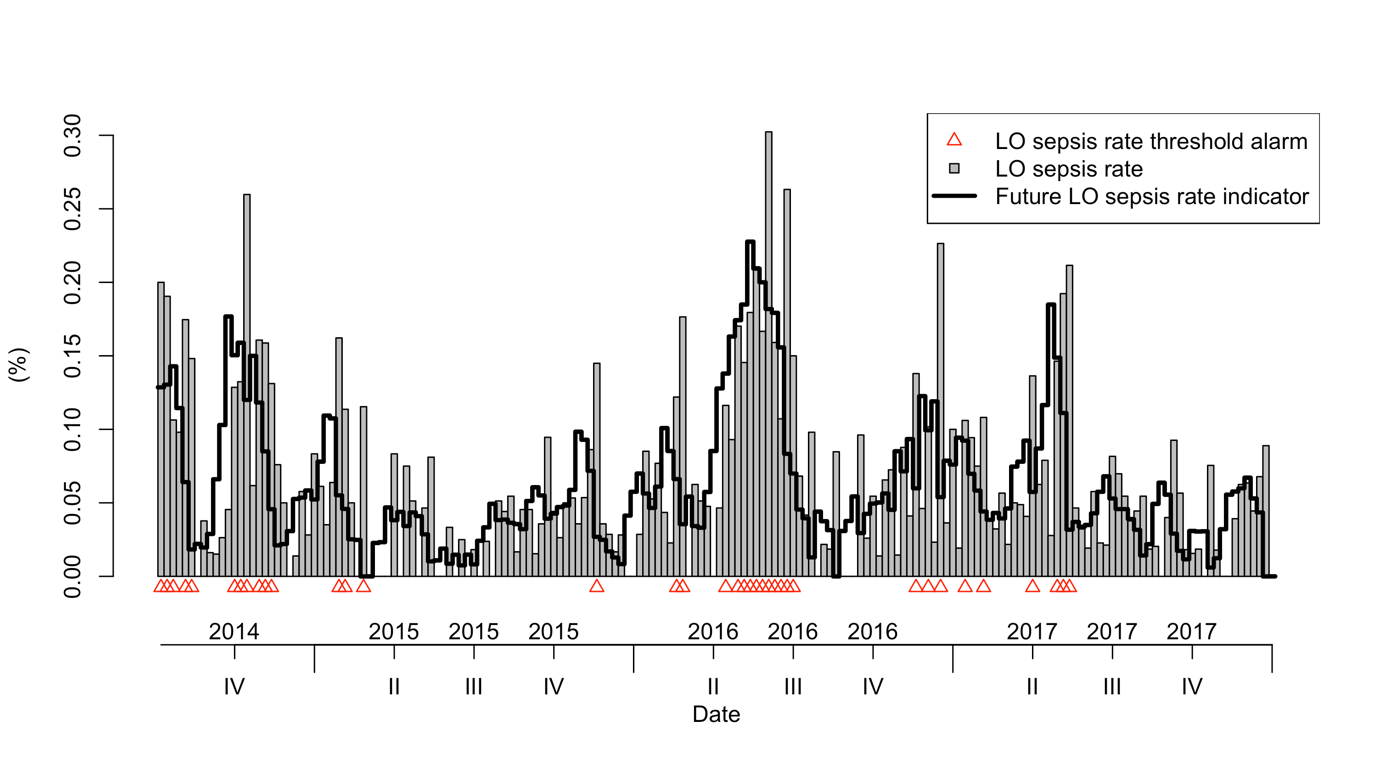 |
| **S10 Figure:** LO sepsis rate threshold alarm (red triangles) and the smoothed future NCU mortality indicator |
| 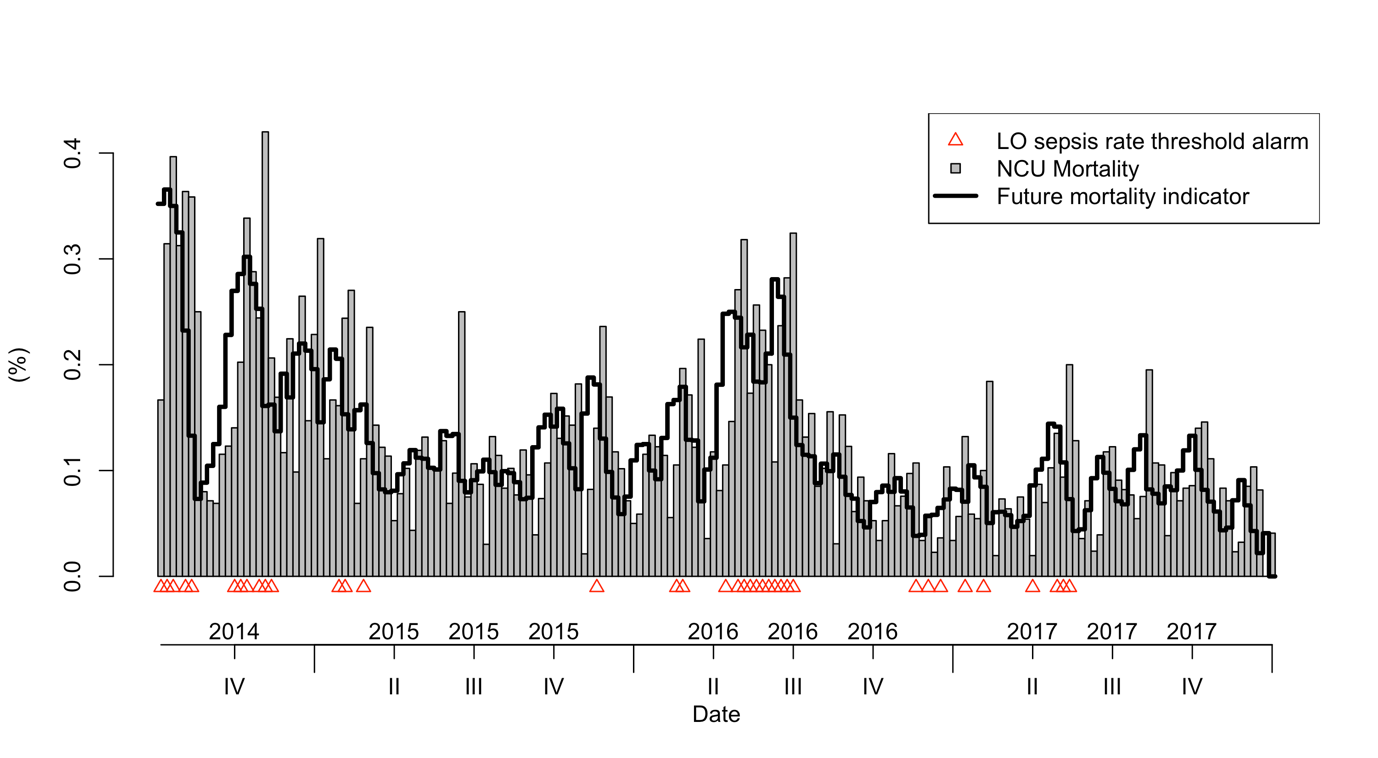 |

| **S11 Figure:** LO sepsis rate threshold alarm (red triangles) and the smoothed GNB positive blood culture indicator |
| --- |
| 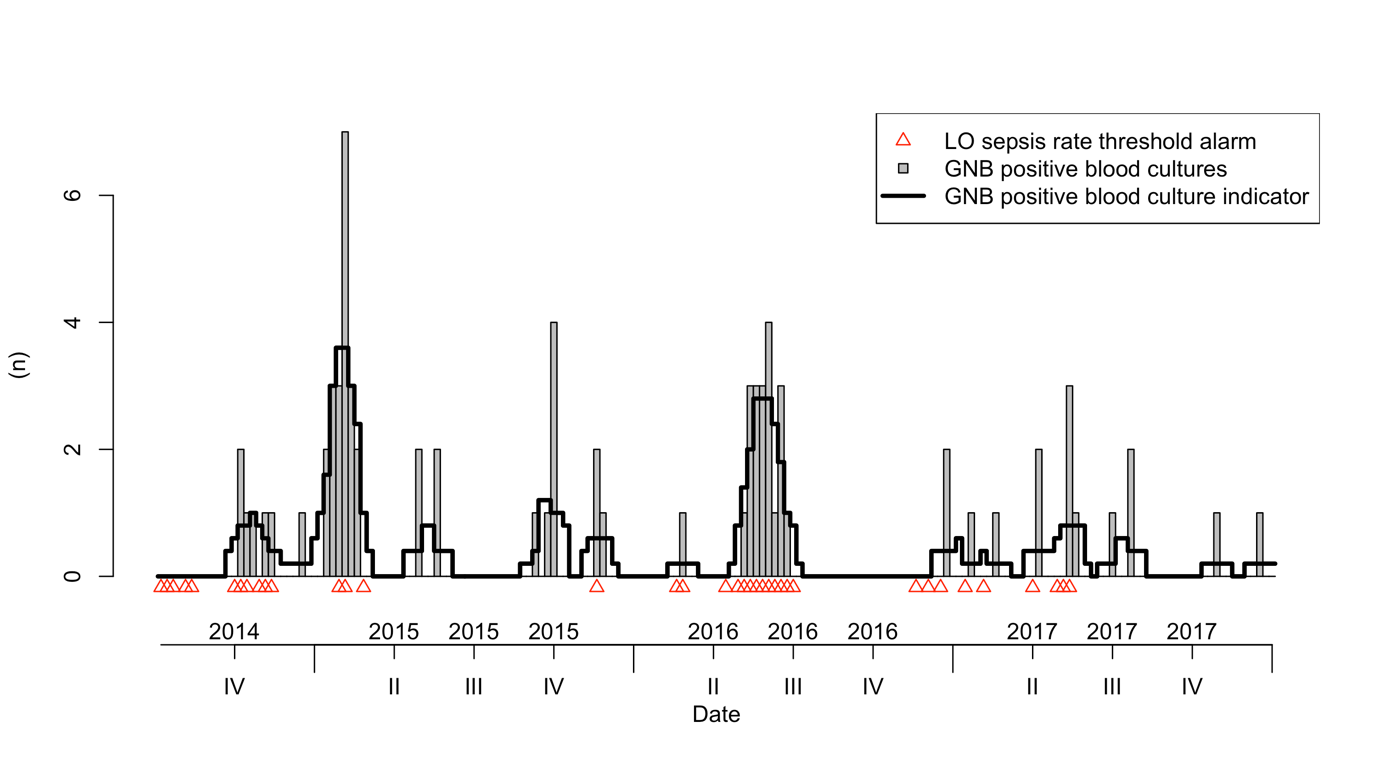 |
| **S12 Figure:** LO sepsis rate threshold alarm (red triangles) and the smoothed LO sepsis rate indicator |
| 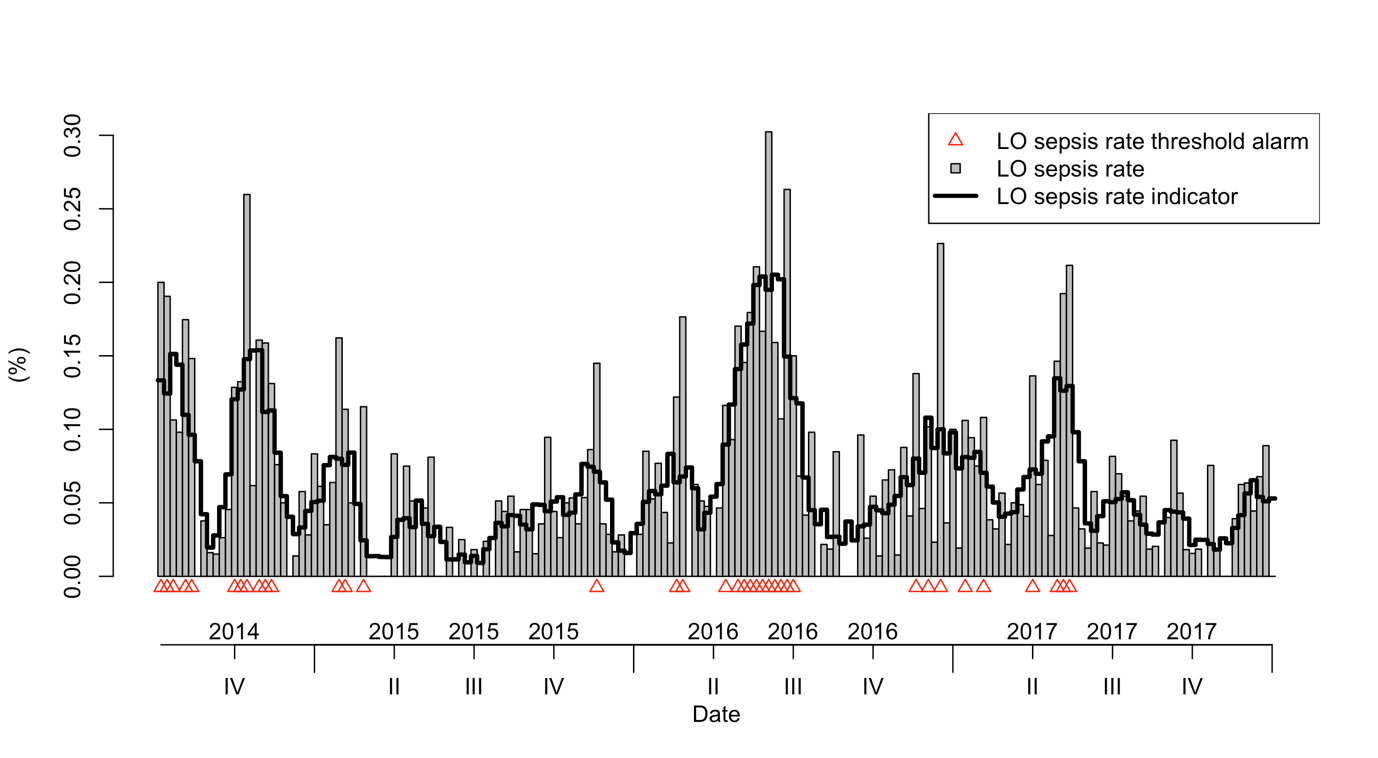 |

| **S13 Figure:** LO sepsis rate threshold alarm (red triangles) and the smoothed mortality indicator |
| --- |
| 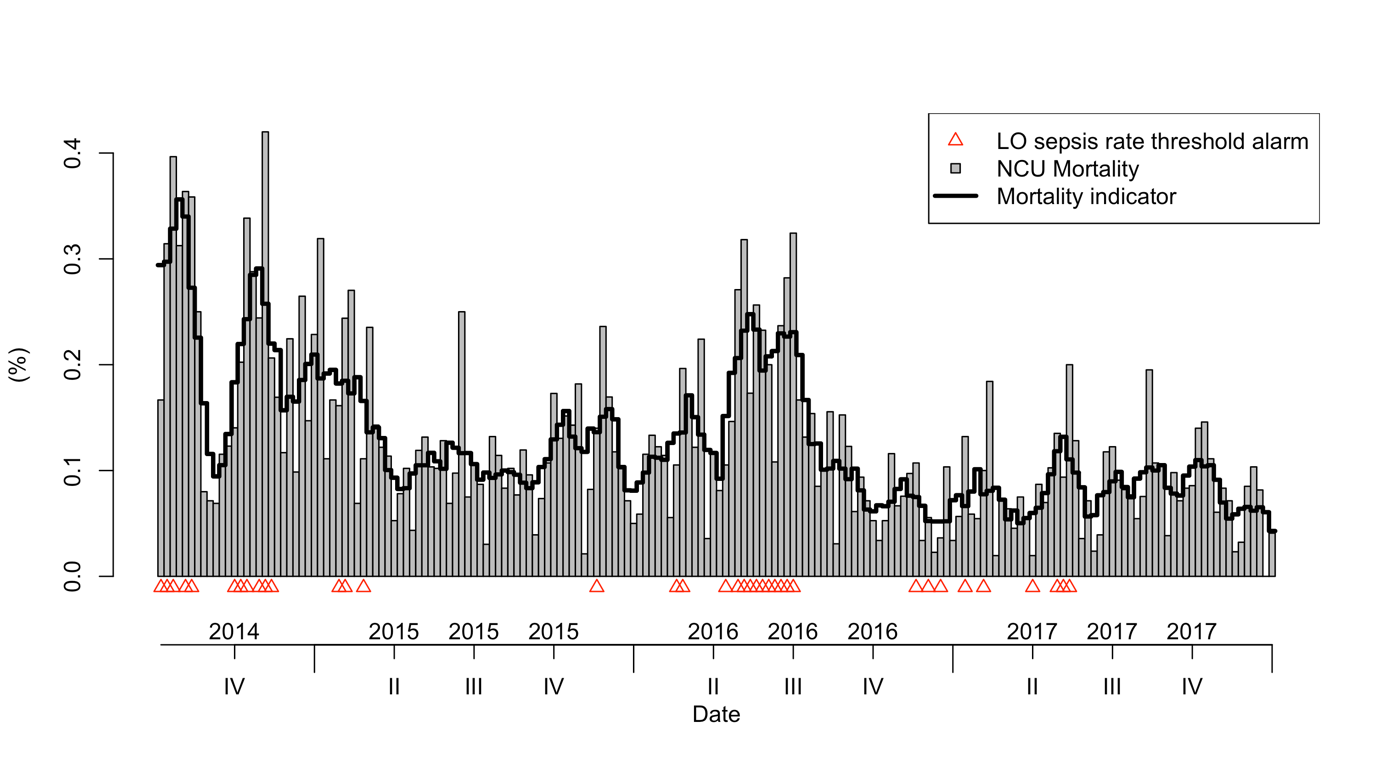 |
| **S14 Figure:** LO sepsis cases threshold alarm (red triangles) and the smoothed future LO sepsis rate indicator |
| 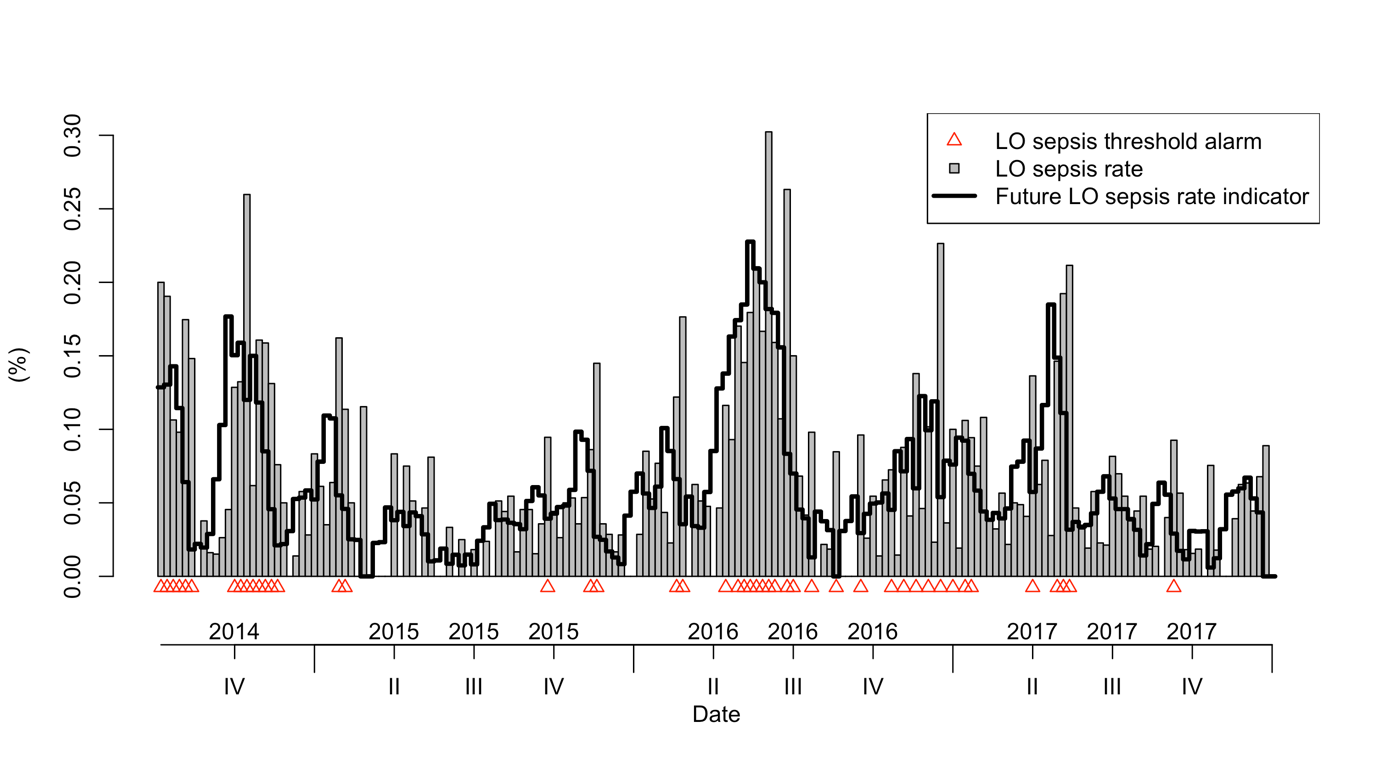 |

| **S15 Figure:** LO sepsis cases threshold alarm (red triangles) and the smoothed future mortality indicator |
| --- |
| 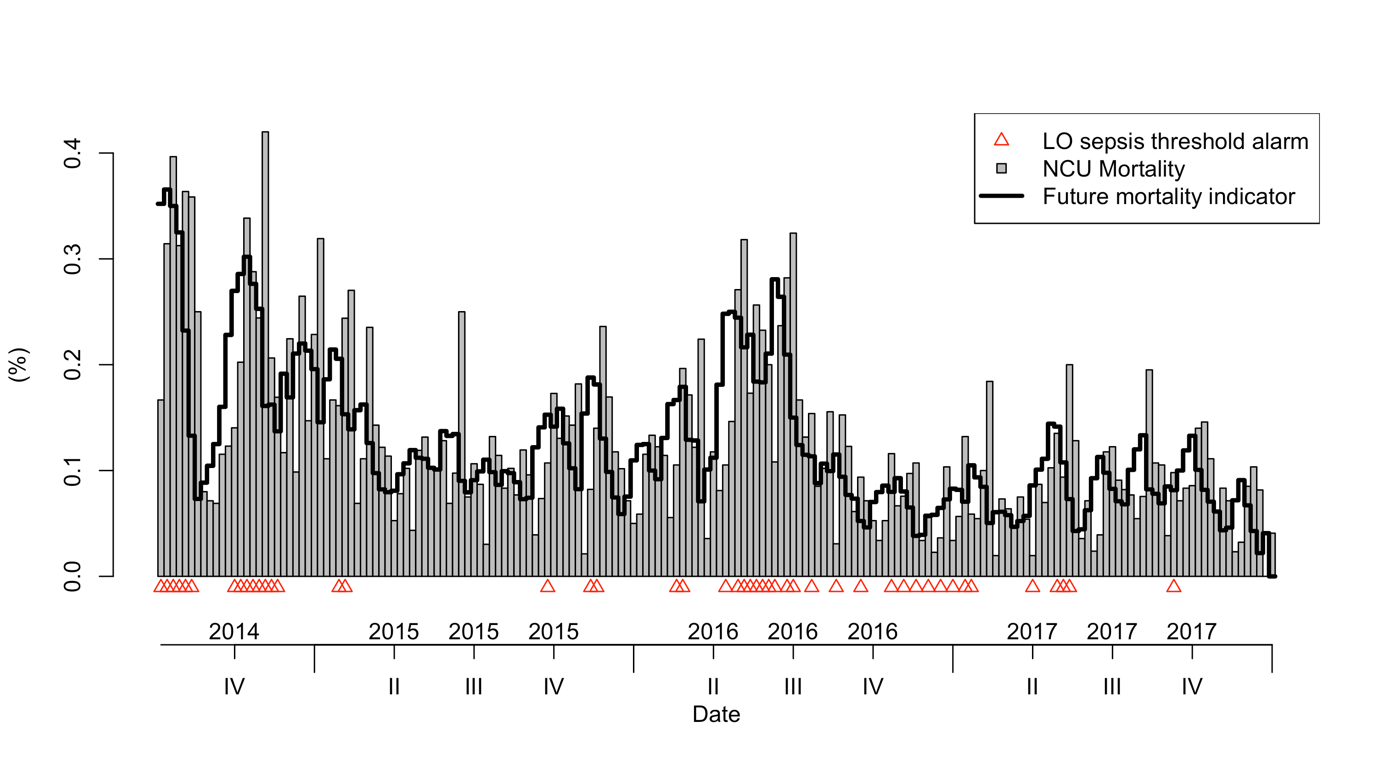 |
| **S16 Figure:** LO sepsis cases threshold alarm (red triangles) and the smoothed GNB positive blood culture indicator |
| 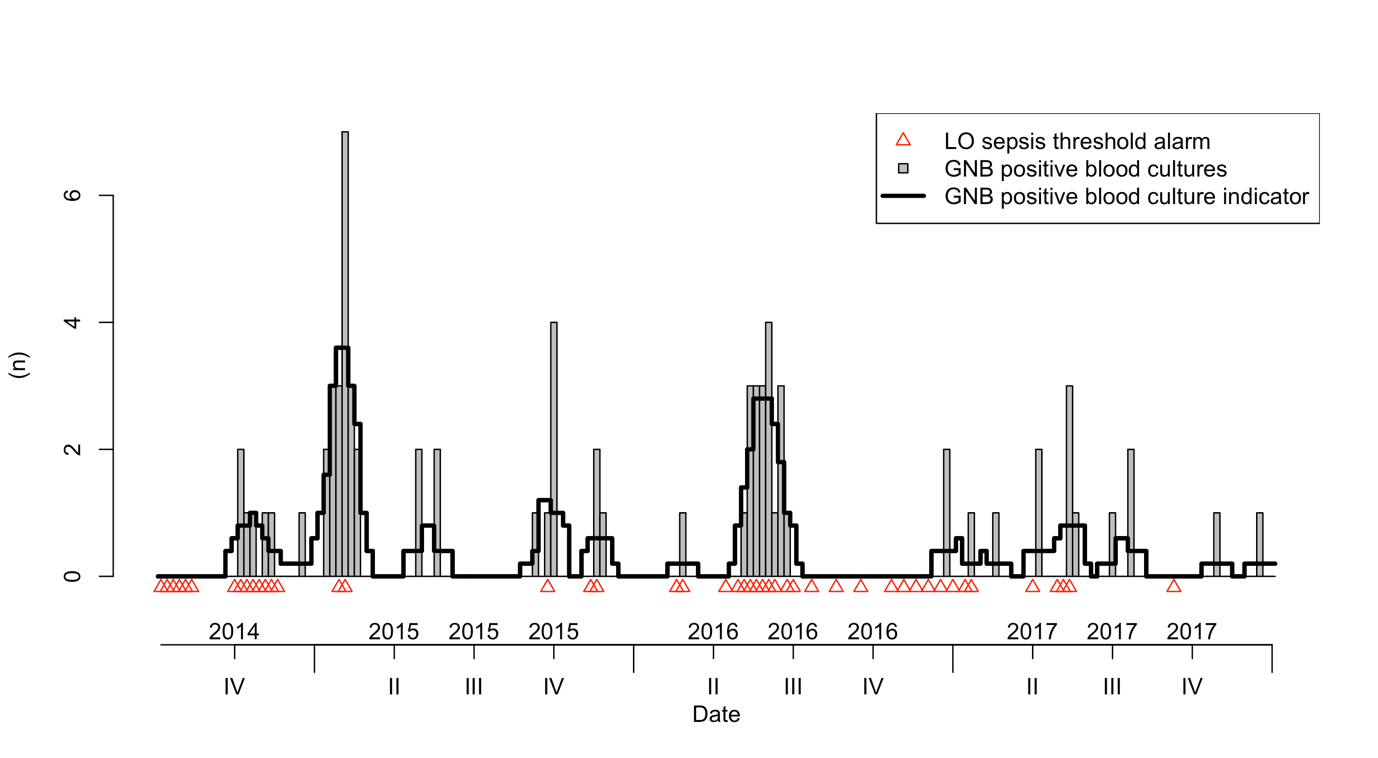 |

| **S17 Figure:** LO sepsis cases threshold alarm (red triangles) and the smoothed LO sepsis rate indicator |
| --- |
| 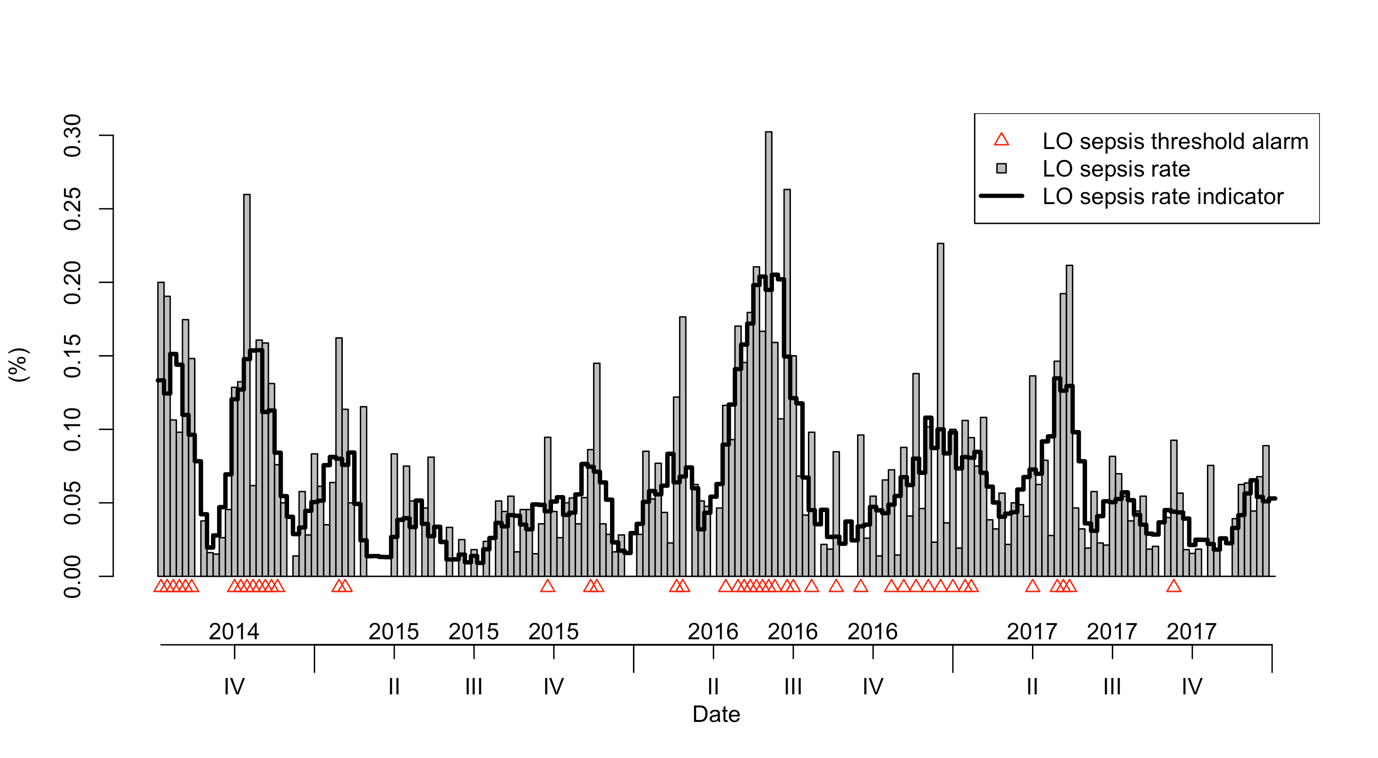 |
| **S18 Figure:** LO sepsis cases threshold alarm (red triangles) and the smoothed mortality indicator |
| 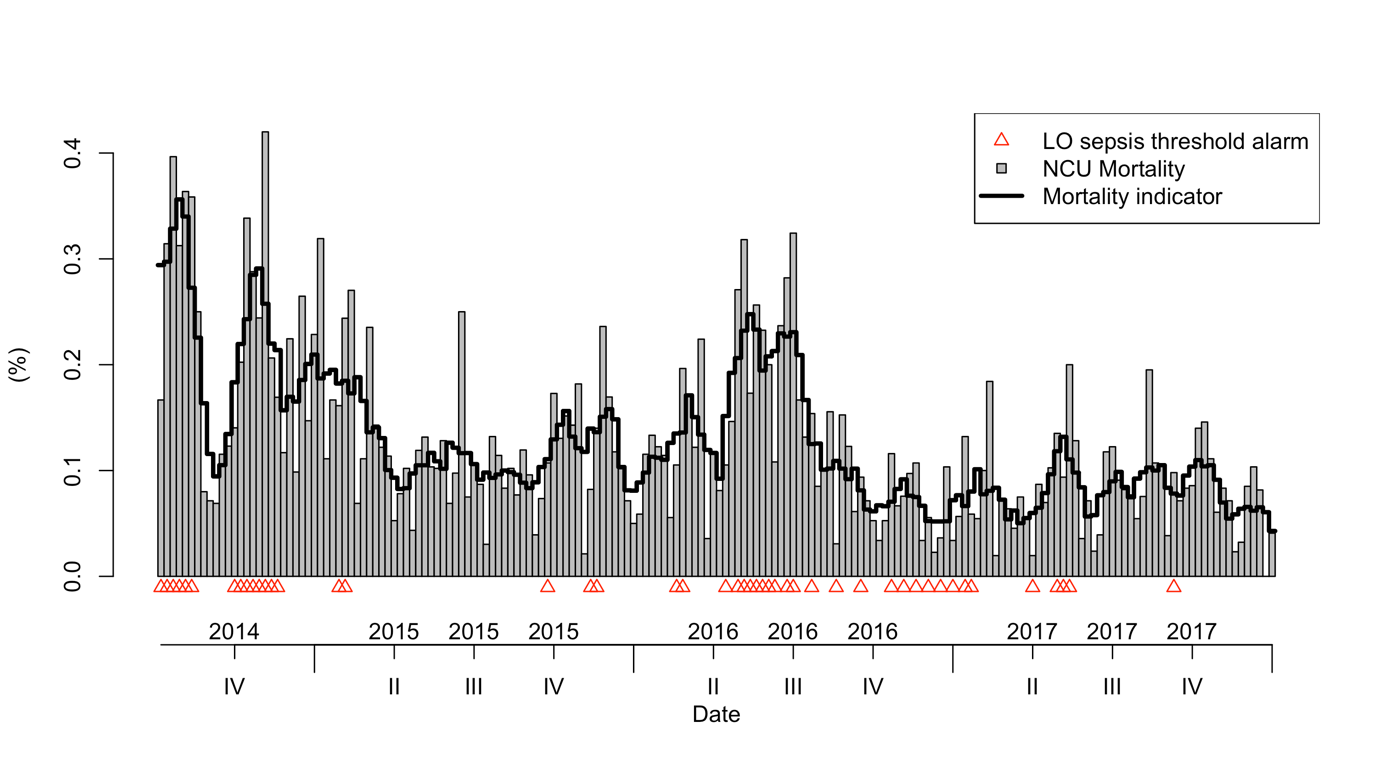 |

| **S19 Figure:** Mortality aberration alarm (red triangles) and the smoothed future LO sepsis rate indicator |
| --- |
| 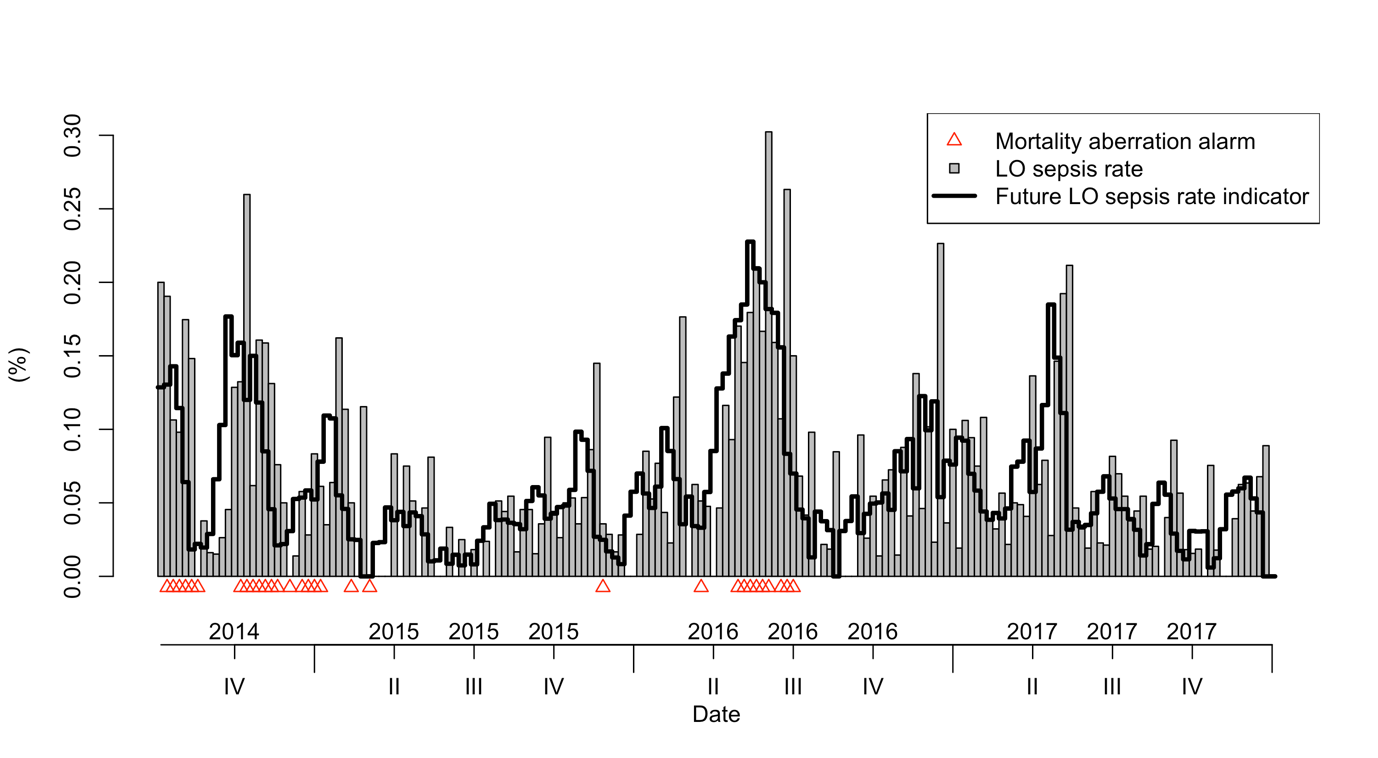 |
| **S20 Figure:** Mortality aberration alarm (red triangles) and the smoothed future mortality indicator |
| 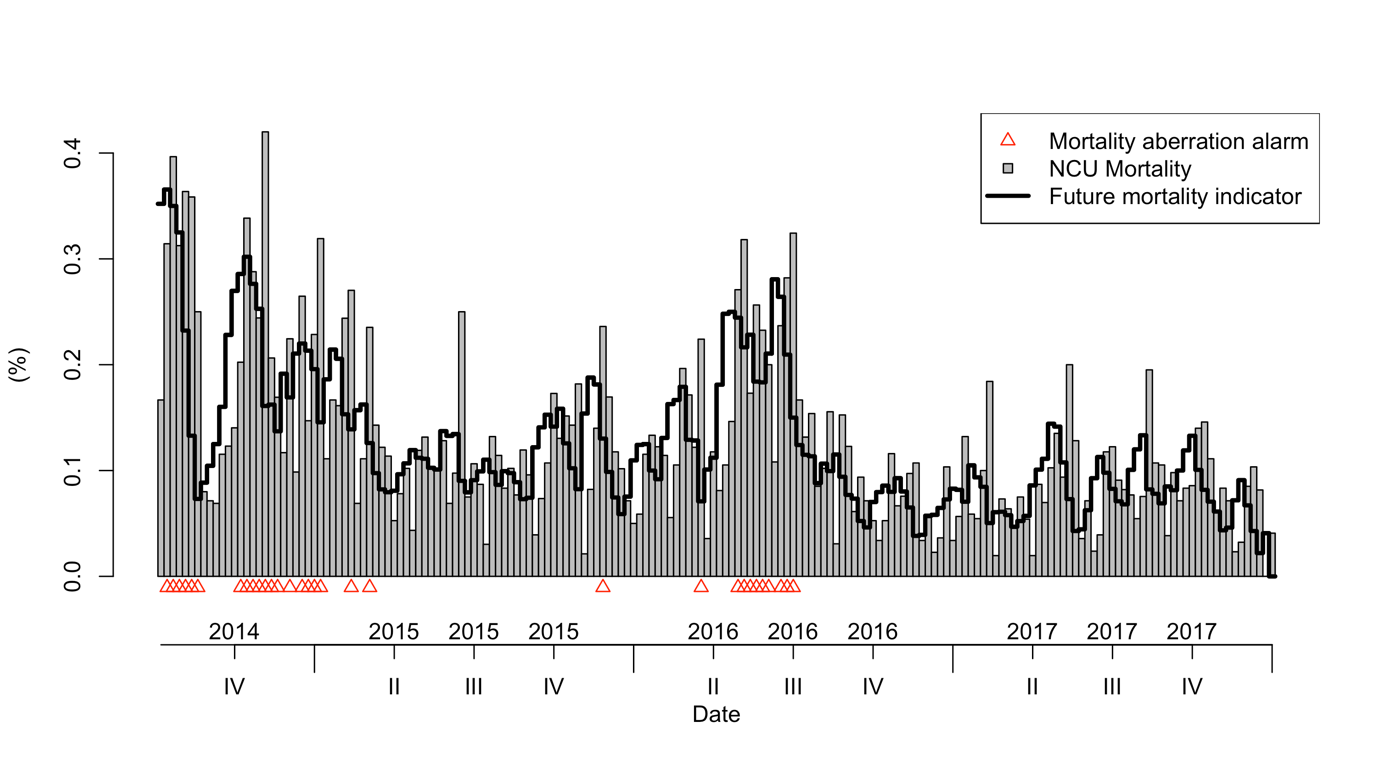 |

| **S21 Figure:** Mortality aberration alarm (red triangles) and the smoothed GNB positive blood culture indicator |
| --- |
| 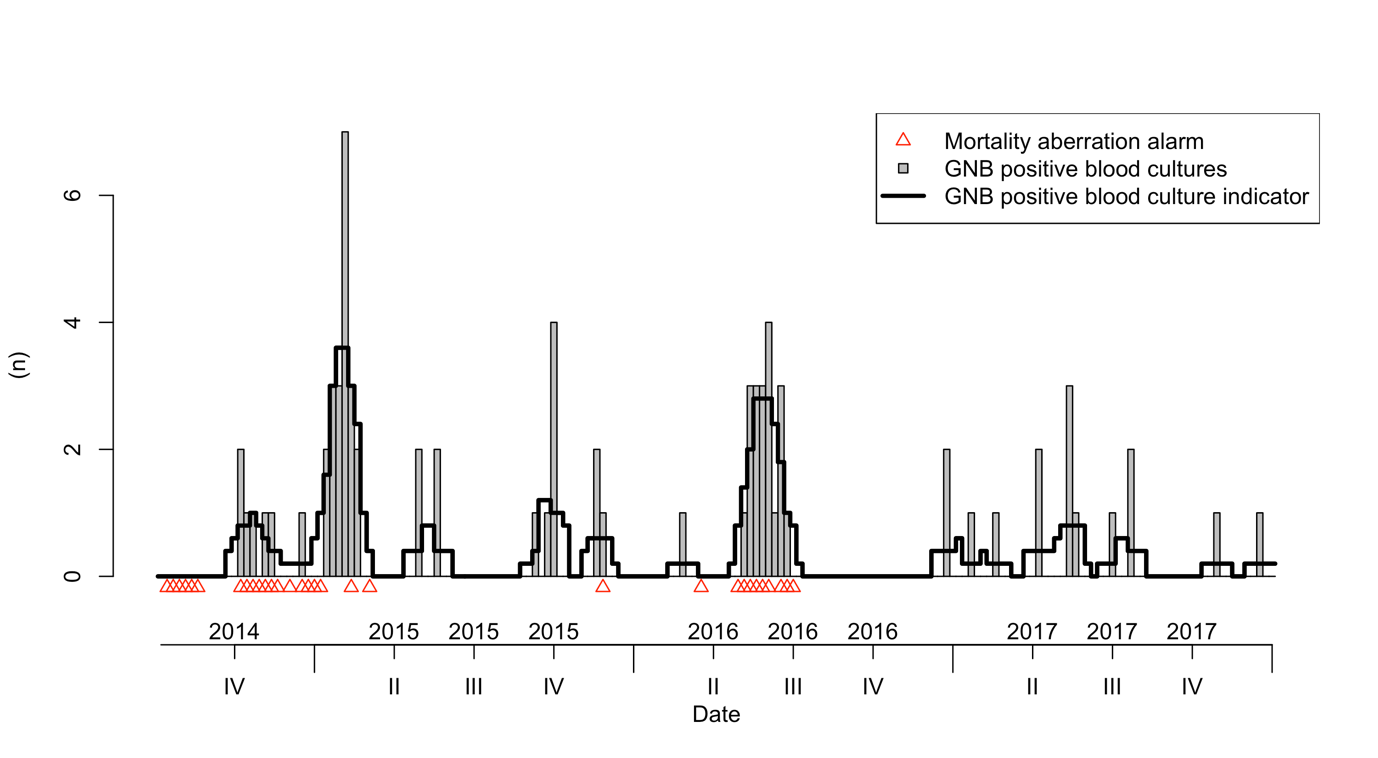 |
| **S22 Figure:** Mortality aberration alarm (red triangles) and the smoothed LO sepsis rate indicator |
| 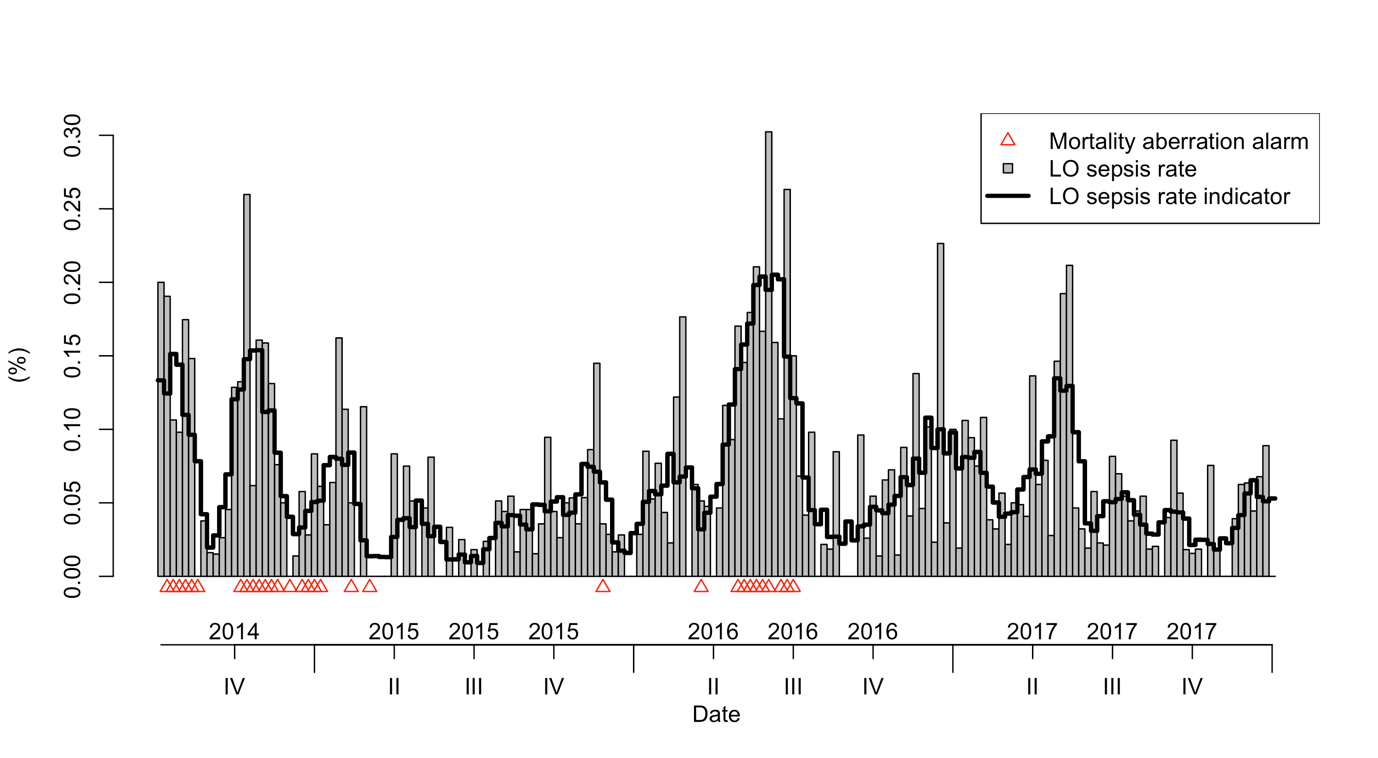 |

| **S23 Figure:** Mortality aberration alarm (red triangles) and the smoothed mortality indicator |
| --- |
| 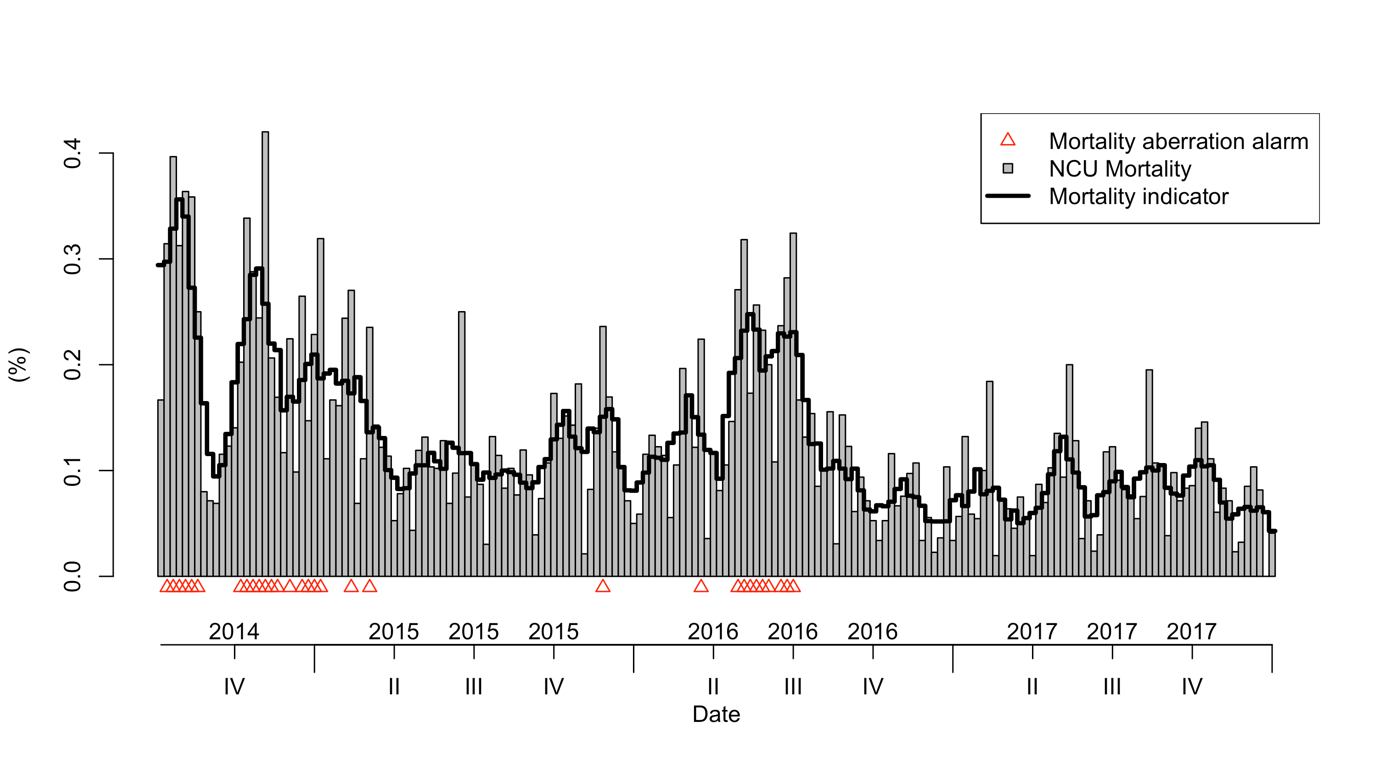 |
| **S24 Figure:** Mortality differential alarm (red triangles) and the smoothed future LO sepsis rate indicator |
| 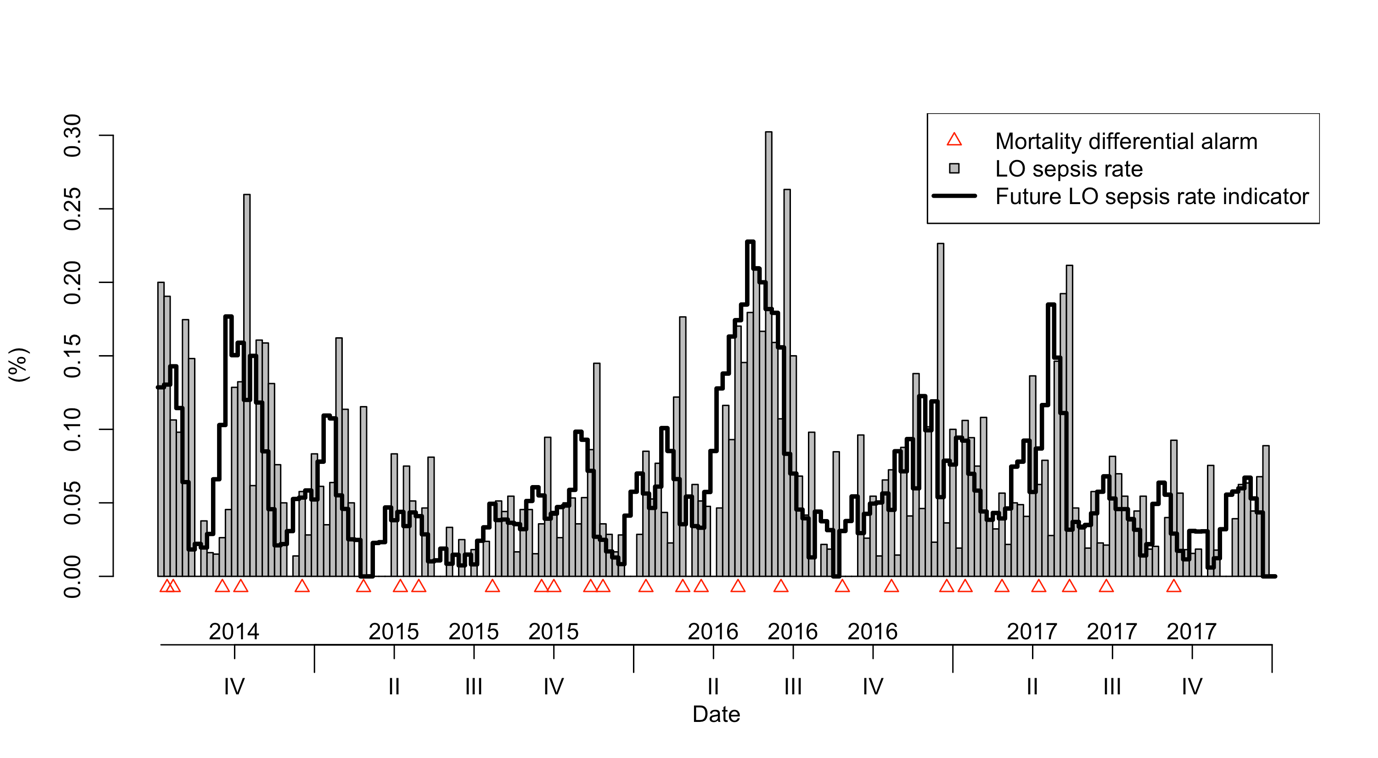 |

| **S25 Figure:** Mortality differential alarm (red triangles) and the smoothed future mortality indicator |
| --- |
| 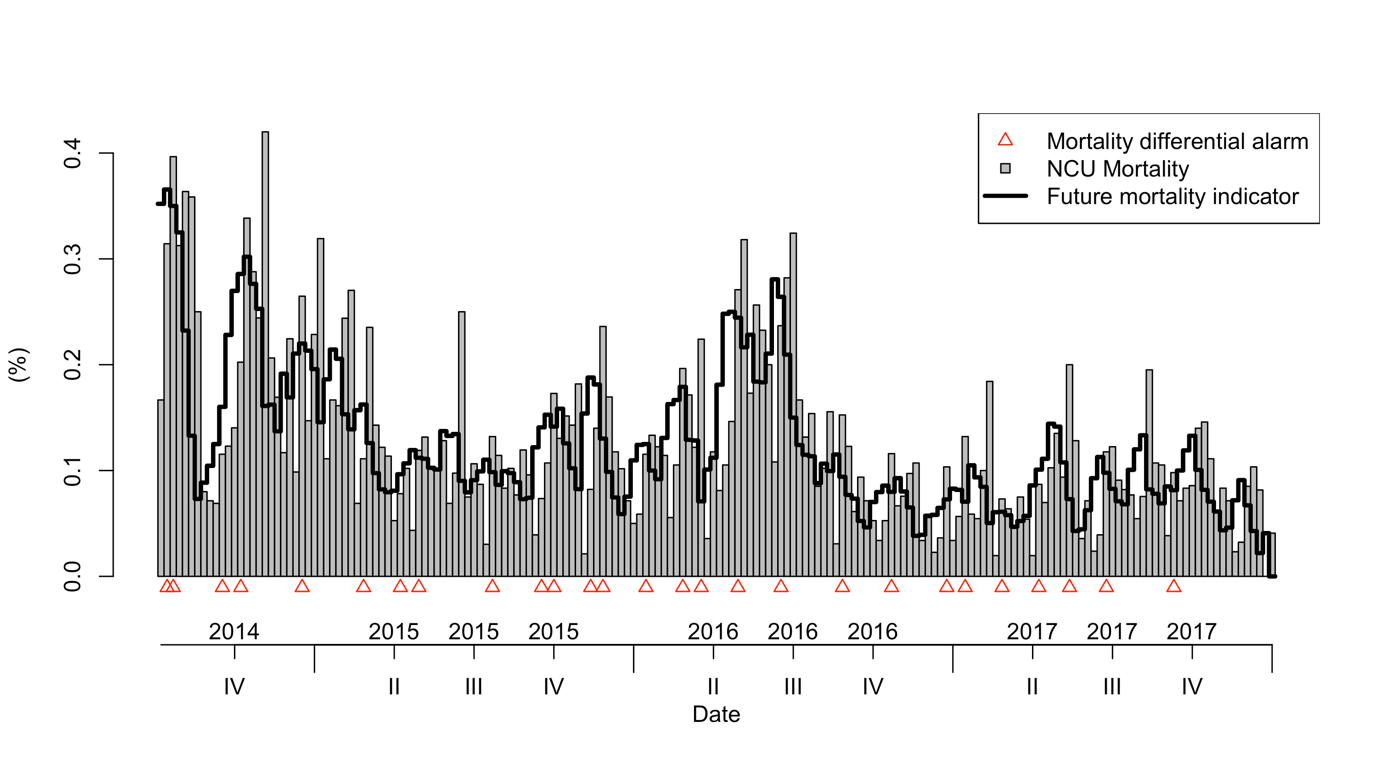 |
| **S26 Figure:** Mortality differential alarm (red triangles) and the smoothed GNB positive blood culture indicator |
| 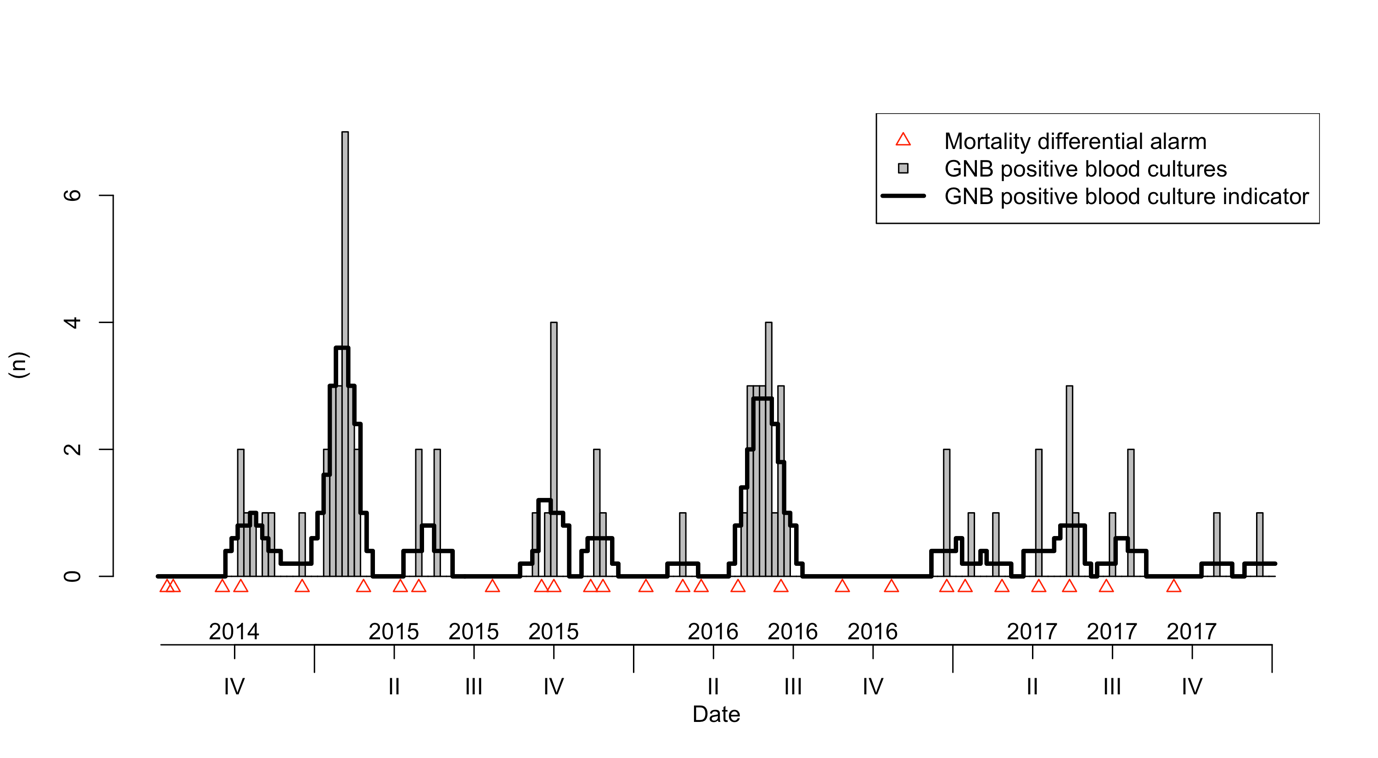 |

| **S27 Figure:** Mortality differential alarm (red triangles) and the smoothed LO sepsis rate indicator |
| --- |
| 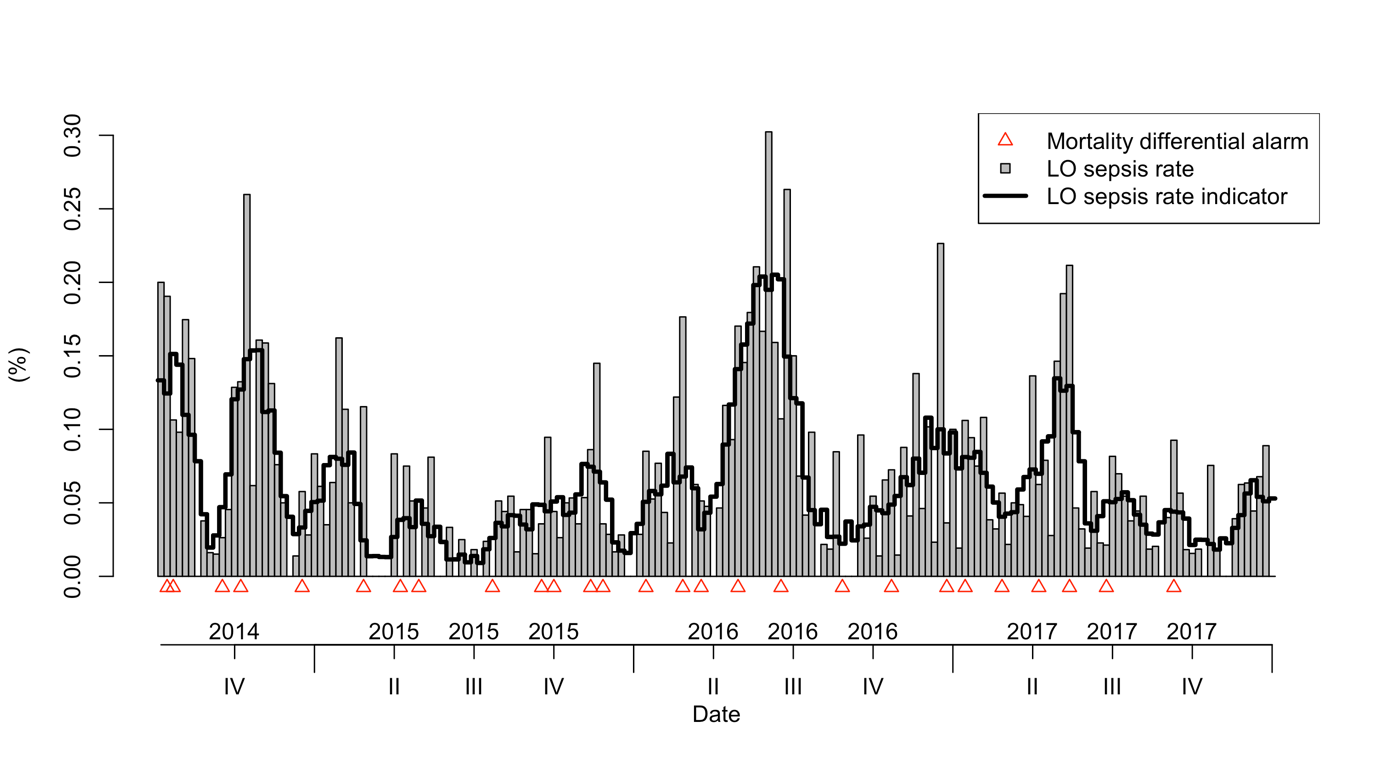 |
| **S28 Figure:** Mortality differential alarm (red triangles) and the smoothed mortality indicator |
| 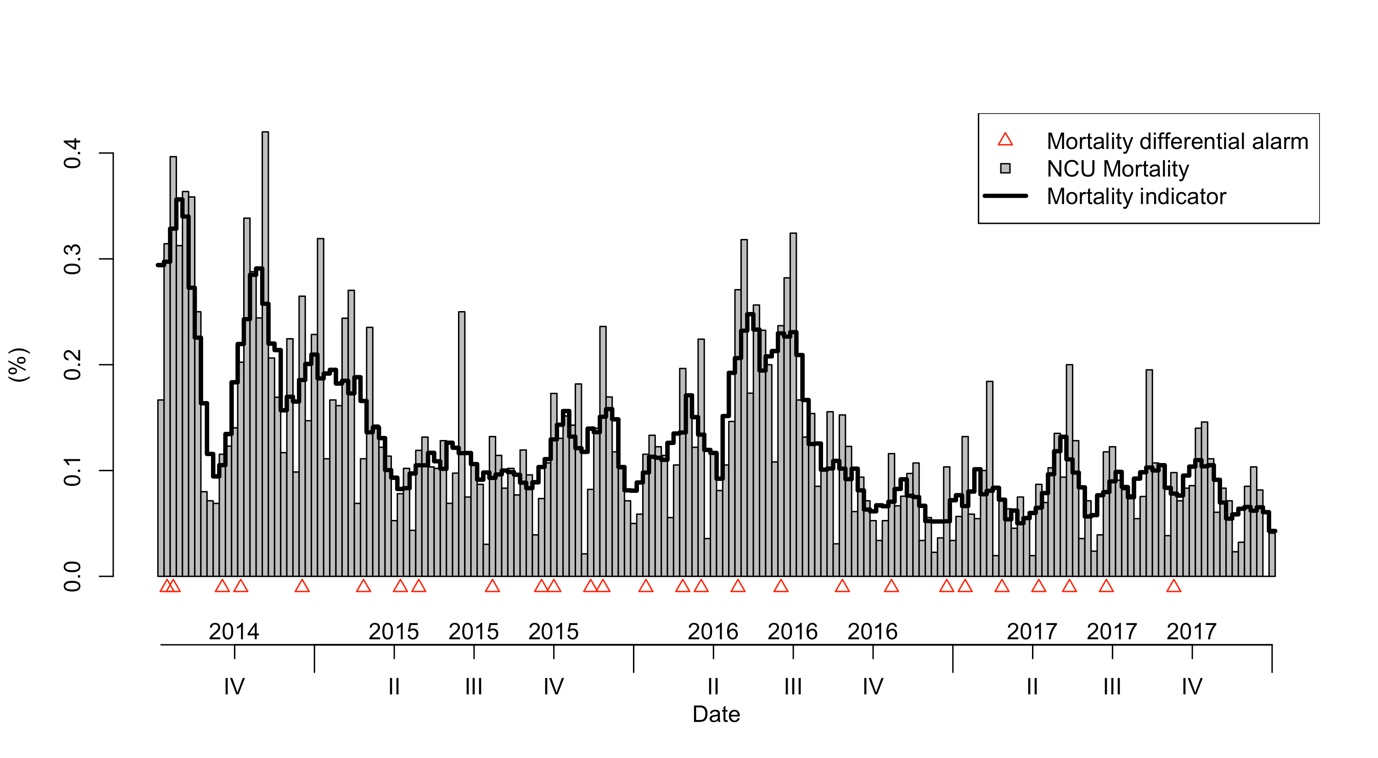 |

| **S29 Figure:** Mortality threshold alarm (red triangles) and the smoothed future LO sepsis rate indicator |
| --- |
| 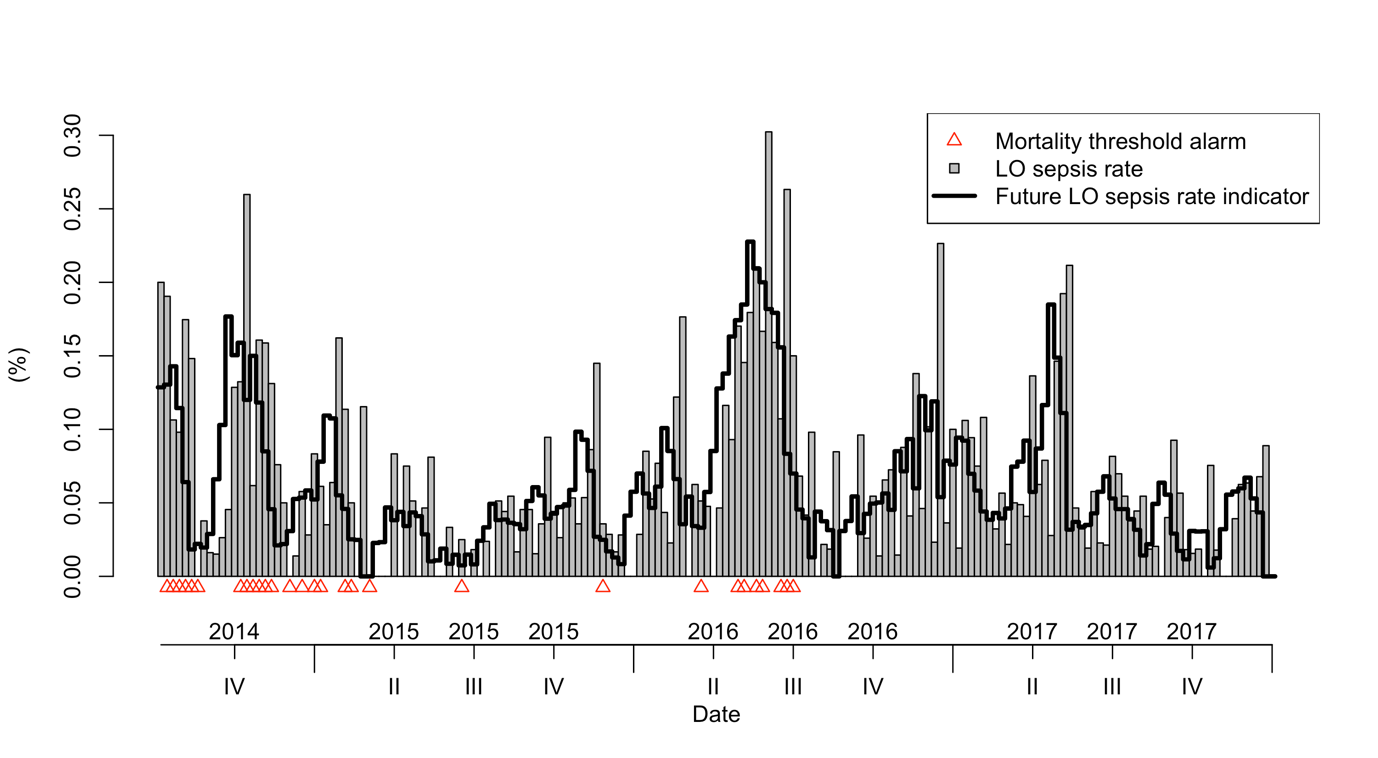 |
| **S30 Figure:** Mortality threshold alarm (red triangles) and the smoothed future mortality indicator |
| 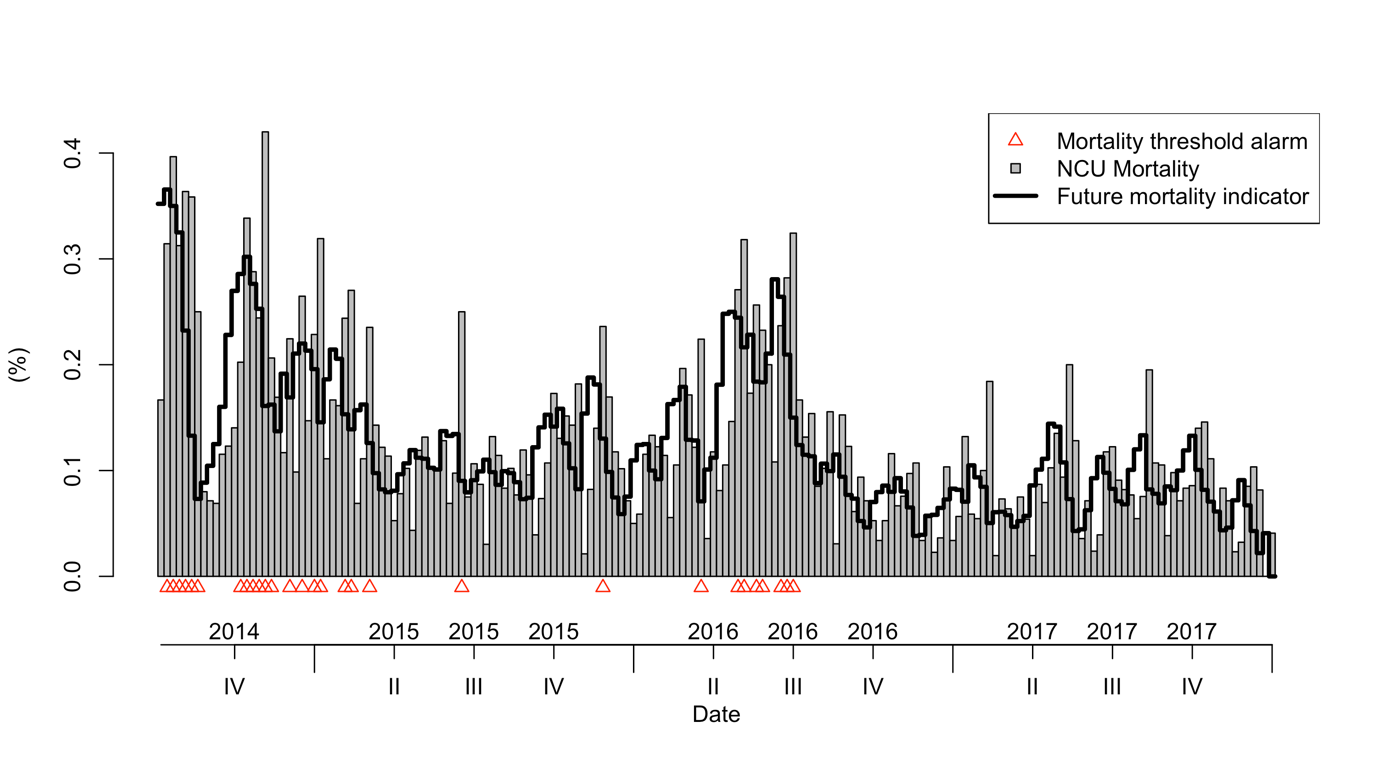 |

| **S31 Figure:** Mortality threshold alarm (red triangles) and the smoothed GNB positive blood culture indicator |
| --- |
| 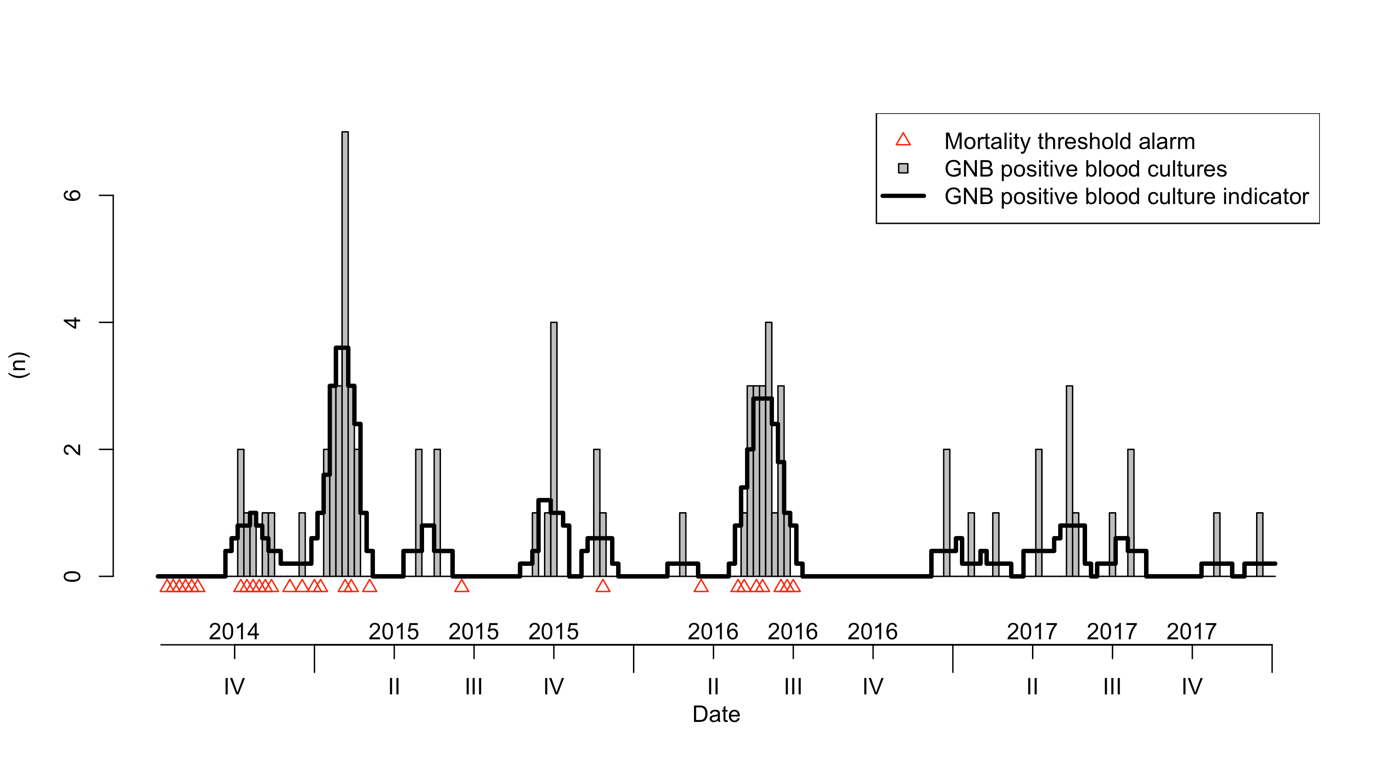 |
| **S32 Figure:** Mortality threshold alarm (red triangles) and the smoothed LO sepsis rate indicator |
| 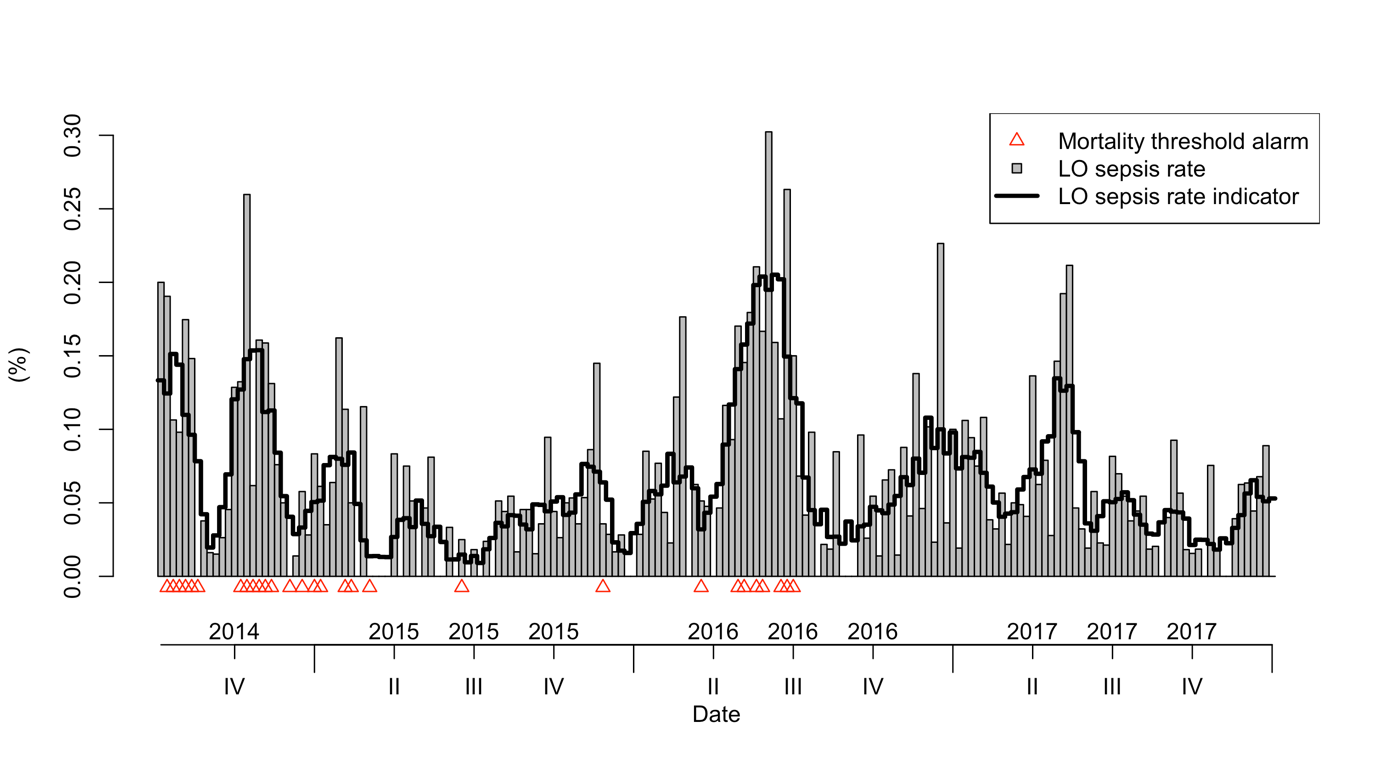 |

| **S33 Figure:** Mortality threshold alarm (red triangles) and the smoothed mortality indicator |
| --- |
| 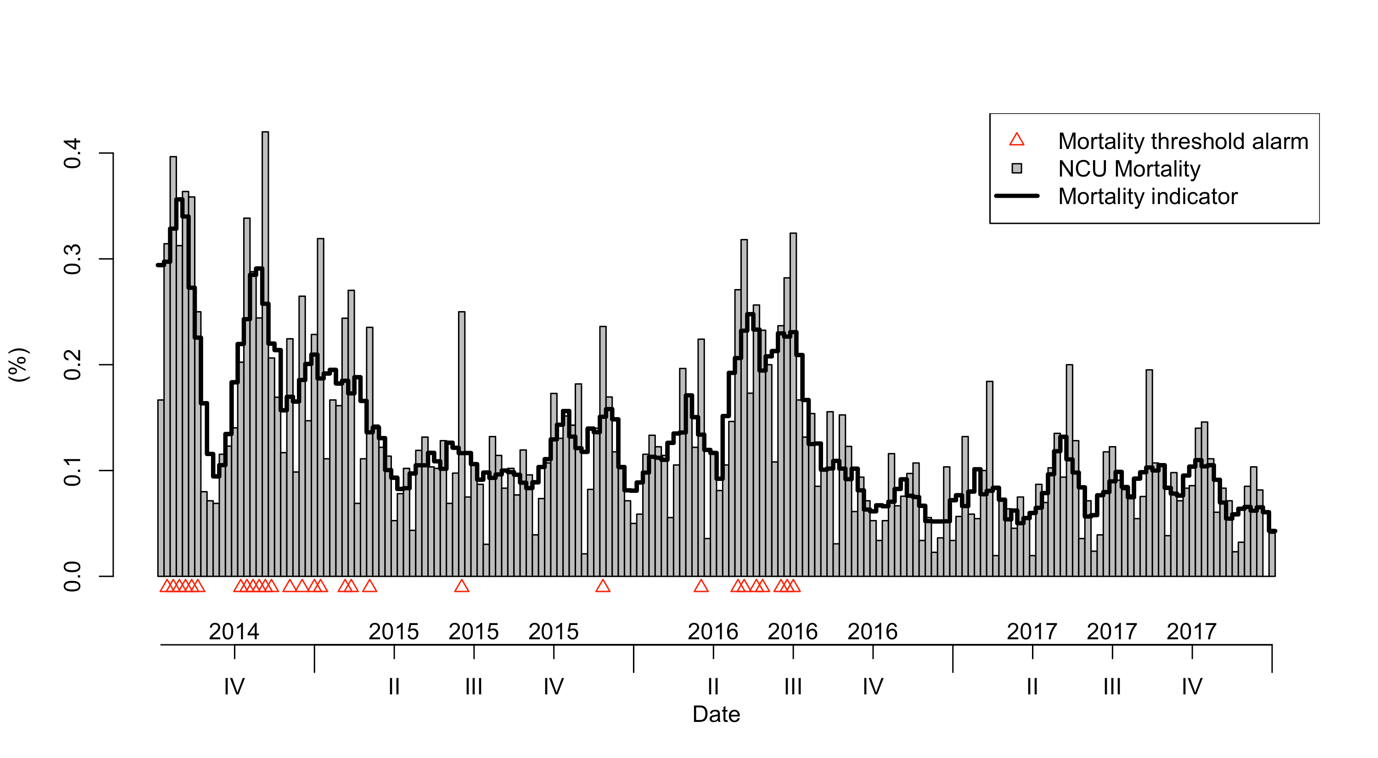 |
